# Diversification and functional expansion of archaeal T4F machineries

**DOI:** 10.1371/journal.pgen.1012139

**Published:** 2026-10-14

**Authors:** Shamphavi Sivabalasarma, Sonja-Verena Albers

**Affiliations:** 1 Molecular Biology of Archaea, Faculty of Biology, University of Freiburg, Freiburg, Germany; 2 CIBSS – Centre for Integrative Biological Signaling Studies, University of Freiburg, Freiburg, Germany; Michigan State University, UNITED STATES OF AMERICA

## Abstract

Archaea have several cell surface structures belonging to the type 4 filament (T4F) superfamily. All share a minimal core assembly system consisting of an ATPase, a platform protein, a filament-forming protein, and a class III signal peptidase. Here, we generated novel MacSyFinder2 models to identify and classify archaeal T4F systems. Our analysis revealed a vast diversity of archaeal T4F, with many organisms harboring one or more assembly machineries. Structure-based phylogenetic analysis showed that the variable N-terminal domain of T4F-related ATPases mirrors the subsystem clustering, indicating that the core machinery has diversified within the archaeal secretion ATPase family through structural innovation. Genome-wide screening for signal peptidase III cleavage site (SPIII) containing proteins revealed the widespread presence of substrate-binding proteins carrying a class III signal peptide. We propose that these binding proteins, with canonical SPIII cleavage sites, functionalize T4F machineries for efficient substrate scavenging, expanding the functional repertoire of archaeal T4F systems beyond their currently characterized roles.

## Author summary

The type IV filament superfamily T4F comprises a broad group of surface structures that are widespread across both archaea and bacteria. While most well-characterized members are bacterial, only a limited number of archaeal T4F systems have been experimentally studied. Here, we expand the known diversity of archaeal T4F loci through bioinformatic and comparative genomic analyses. Our results show that these systems are far more diverse and versatile than previously appreciated, often exhibiting specialized functions. Structural diversification of the ATPase machinery likely played a key role in driving the functional diversification of T4F systems. Together, these findings deepen our understanding of how archaea adapt and persist in diverse environments, highlighting their surface structures as essential tools for communication, adhesion, and nutrient acquisition.

## Introduction

Archaea harbor versatile cell surface structures that decorate their cell envelope many of which belong to the type IV filament superfamily (T4F) [1]. This T4F superfamily includes surface structures in archaea and bacteria, such as the type IV pilus machinery or the archaeal motility machinery, the archaellum. Its members evolved from a common ancestor with a minimal core machinery: a prepilin/archaellin peptidase, a filament forming subunit and a membrane integral platform protein in interaction with an ATPase (Fig 1). The prepilin peptidase is a class III signal peptidase essential for processing filament subunits that are inserted at the base of the platform protein [2–4]. The hexameric AAA + ATPase provides energy for filament assembly and function [5–9] (Fig 1C). Each machinery serves a specific function through incorporation of additional accessory genes, as, e.g., the archaellum that incorporated stator complexes by duplicating the filament subunit [10]. Several archaeal T4F members have been studied *in vivo*, as in *Sulfolobus acidocaldarius*, which encodes multiple T4F machineries. The archaellum (7–11 genes) mediates motility and swimming, while the Aap pilus (Archaeal adhesion pilus), a 5 gene cluster, supports twitching motility, adhesion, and biofilm formation [6,11]. The UV-inducible pilus, a T4F specialization, enables cell recognition and DNA exchange for recombination and repair [8,12], and some methanogens encode the Epd pilus that contributes to cell adhesion [13,14].

**Fig 1:**
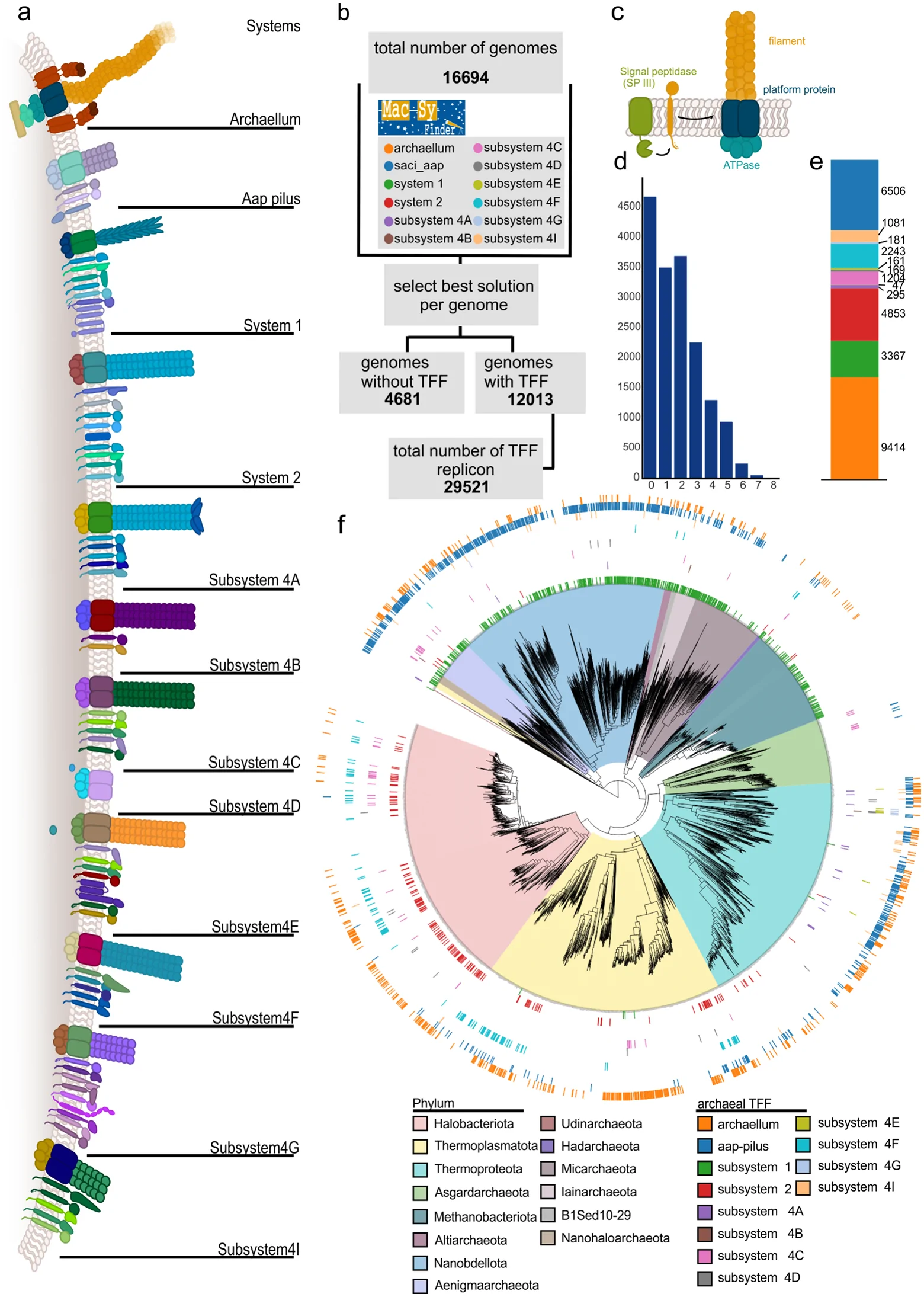
Diversity of archaeal T4F systems. **a** Schematic of all 12 archaeal subsystems, each with a MacSyFinder model. b Workflow for identifying archaeal T4F systems using a dedicated model for each subsystem. **c** Core T4F machinery components of an archaeal T4F consisting of a class III signal peptidase, a platform protein, an ATPase and a filament forming protein. **d** Number of genomes encoding multiple T4F loci. **e** Stacked bar graph of the number of identified T4F per subsystem. Colours are as in **f**. GTDB derived phylogenetic tree of archaea with the presence of each T4F subsystem per organism.

While all machineries share orthologous core components, specific accessory components are found in each system. The archaellum harbors proteins for torque generation while the UV pilus includes DNA processing proteins in close gene neighborhood [15,16]. Previous analyses of putative archaeal T4F machineries clustered them into 13 subsystems differing in gene locus composition and accessory components [17]. Here we expanded this analysis on the now vast number of archaeal genomes and found T4F loci to be widespread in archaea. MacSyFinder2 (Macromolecular systems finder) was used for a semi-automated search of archaeal T4F machineries, allowing us to discriminate archaea specific systems and to understand the composition of each subsystem [18]. Structure-based phylogenetic analysis of the ATPase the core component of the T4F superfamily using FoldTree [19] showed that they cluster according to subsystem, revealing extensive diversification within the archaeal secretion ATPase family that is observed in the structural variety of the N-terminal domains.

These surface structures may contribute to efficient functionalization of the cell surface, aiding in substrate binding, cell adhesion, cell-cell recognition, and intracellular communication. Indeed, screening archaeal signal peptides for SPIII identified substrate binding proteins that are widespread and processed like T4F subunits, as previously observed for the bindosome in *Saccharolobus solfataricus* [20,21]. We propose that certain T4Fs act as the core machinery for incorporating substrate-binding proteins into pilus filaments, ensuring efficient nutrient and substrate uptake expanding the evolutionary and functional landscape of the T4F superfamily.

## Results

### Widespread presence of archaeal T4F clusters in genomes

In a prior study archaeal T4F systems were classified into 13 subsystems based on sequence similarity of their ATPases [17]. Each system was defined by a specific set of co-localized genes encoding core machinery and accessory components as identified and defined in Makarova et al.,2016. Homologs of each component were grouped into archaeal cluster of orthologous genes (arCOG) (S1 Table, Fig 1a). The arCOGs per system were extracted from the Eggnog database and used to build MacSy-generated models per archaeal T4F system, facilitating the detection and differentiation of T4F (S2 and S3 Tables). We generated an internal database of 16,694 archaeal genomes (as of May 2025) and screened for T4F systems with MacSyFinder2 [18], using separate models for archaellum, system 1, system 2, aap pilus and subsystems 4A-I (Fig 1a and 1b).

We focused on ordered replicons T4F to resolve subsystems by gene co-localization, identifying 29,521 replicons encoding bona fide T4F machineries (Fig 1b, S1 Table). This search found 12,013 genomes encoding at least one T4F system. Although some genomes do not encode a T4F within one locus, several harbor multiple T4F loci (Fig 1d). The archaellum locus is often accompanied by additional T4F loci. Most genomes encode one or two systems, while some harbor up to 8 complete T4F systems (Fig 1d). To assess the distribution of archaeal T4F subsystems, we mapped the detected ordered replicons onto the Genome Taxonomy Database (GTDB)- derived phylogenetic tree of representative phyla [22] (Fig 1f). The subtypes, as previously defined [17], comprise subsystems specific to the archaellum, the Ups pilus, Methanobacteriati-specific Halo and Epd pilus, and several functionally unclassified subsystems. Additionally, we introduced a subsystem for the Aap pilus (formerly part of subsystem 4A) to distinguish experimentally shown retractable pili from other nonretractable structures. Most classified T4F subsystems correspond to archaella (9414) or Aap pilus (6506) (Fig 1e). System 4 comprises a plethora of T4F machineries with various locus organization and accessory components. These are divided into eight subsystems (4A-G and 4I) based on the specific accessory components (Fig 1a) [17]. Members of system 4 occur in roughly 2/3 of analyzed replicons and are widespread in Archaea (Fig 1e). They frequently occupy multiple T4F loci in genomes, as seen in several halophiles and Thermococcales, with various sets of co-localized pilins and accessory components (Fig 1d and 1e, S1 and S3 Tables). Overall, archaeal T4F are widespread in most phyla except the Prometheoarchaeati (formerly known as Asgard archaea) and Nanobdellati (formerly known as DPANN archaea). In contrast, members of the Thermoproteota are rich in T4F systems often encoding several T4F machineries per genome. Methanobacteriati (formerly known as euryarchaeota) harbor multiple systems, typically belonging to system 2, system 1 or subsystem 4C, the latter likely a halophile-specific T4F machinery (Fig 1a,1e and 1f). System 1 is predominantly found in methanogens, a few Nanobdellati archaea, and members of Nitrososphaerota (formerly known as Thaumarchaeota) (Fig 1f).

### T4F-related genes are rarely found within one locus in Prometheoarchaeati and Nanobdellati

In contrast to most Archaea of other superphyla, members from the Prometheoarchaeati and Nanobdellati rarely showed an ordered T4F locus. Hence, an additional screening of all available genomes of these two lineages in unordered mode using MacSyFinder2 was done, which detected T4F components with a scattered localization in the genome (S4 Table, S10 Fig). This identified 677 Prometheoarchaeati and 2942 Nanobdellati genomes containing components for a T4F system (S4 Table). Most encode the core components of a T4F machinery including homologs for an ATPase, platform protein varying numbers of pilins and a signal peptidase indicating the expression would produce an assembled pilus machinery with a surface filament. Notably, a few Heimdallarchaea encode such a simple T4F containing only core machinery components. *Ca.* Lokiarchaeum ossiferum encodes an archaellum [23]. Similarly, recently characterized Hodarchaeales *Ca. Margulisarchaeum peptidophila* and *Ca. Flexarchaeum multiprotrusionis* as well as *Ca. Nerearchaeum marumarumayae*, display surface structures likely belonging to the T4F superfamily [24,25]. The locus organization is more scattered in *Ca. Flexarchaeum multiprotrusionis* compared to *Ca. Margulisarchaeum peptidophila* which has putative archaellin, stator and platform protein genes encoded in close gene neighborhood (S10 Fig, S4 Table).

Prometheoarchaeati have a rather fragmented distribution of T4F genes where the core components are only rarely found in gene neighborhood. In Nanobdellati, the ATPase and platform protein are found close by (within 5 genes) or even adjacent while pilins and subsystem specific components are more scattered. We tried to identify which subsystem can be assigned to the unordered T4F loci. For most, the identity of the T4F system is ambiguous, as only one or two subsystem-specific components were found (S5 Table). In Prometheoarchaeati, 139 genomes encode likely an Aap-like pilus as characterized in Sulfolobales, including AapAB pilin homologs belonging to arCOG03871, often co-occurring with the Fe-S cluster protein (arCOG07320). 100 prometheoarchaeal genomes likely encode an archaellum. While some, such as *Ca.* L. ossiferum, encode an archaellum in an ordered locus, most seem to have a scattered organization of the archaellum components. What is striking is the presence of ArlFG homologs (arCOG01822, arCOG01824) as well as multiple ArlH homologs (arCOG04148) and some have an ArlCDE/ArlX or a CheF homolog. Together, this suggests that some Prometheoarchaeati have an archaellum integrated into the enigmatic prometheoarchaeal cell envelope via its stator proteins, and that CheF links the archaellum to the chemotaxis system. In Nanobdellati, 167 genomes have an archaellum. The scattered organization has already been described for some, such as *Nanobdella aerobiophila* and *Nanoclepta minutus* [26,27].1990 genomes encode pilin subunits specific to system 1, likely resembling the methanogenic Epd pilus. In both Prometheoarchaeati and Nanobdellati, subsystem 4 gene loci are sparsely distributed, though most (17 in Prometheoarchaeati and 25 in Nanobdellati) encode components specific to subsystem 4D (S4 and S5 Tables). Subsystem 4D is likely a remnant T4F and because its components are scattered across the genome, they are unlikely to act together.

### Analysis of subsystem specific ATPases reveal variable N-terminal domains

All archaeal T4F-related ATPases belong to the secretion ATPases (AAA + ATPases of the VirB11 type), which also includes the bacterial secretion systems and archaeal T4F machineries [1,17]. The archaeal secretion ATPases is organized into four arCOGs (arCOG01818, arCOG01817, arCOG01819, and arCOG05609) (S2 Table), the last of which is specific to subsystem 4G. We predicted the structures of ATPases of high-confidence system clusters to build a structure-based phylogenetic tree with FoldTree [19]. The resulting clustering largely mirrors the subsystem organization. Archaellum-related ATPases and those of subsystems 4F, 4G, and 4I form distinct clusters, reflecting evolutionary diversification (Fig 2a). Since these ATPases separate from the archaellum ATPase, the corresponding systems are likely unrelated to motility and instead likely serve alternative roles (Fig 2a). In contrast, system 2, Aap-related ATPases, and some subsystems cluster together, indicating similar structural arrangement and possibly related mechanisms. This separation points to evolutionary diversification of the core machinery driven by the incorporation of accessory components (Fig 2a). Strikingly, while the core domains, including the C-terminal ATP-binding domain, are conserved, additional N- and C-terminal domains are present, specific to each subsystem, suggesting that the core components have adapted to their distinct accessory proteins. The predicted structures of the secretion ATPases from the two model organisms, *Saccharolobus solfataricus* and *Haloferax volcanii*, were compared (Fig 2b). Both encode multiple T4F systems next to the archaellum loci [6,8,11,28,29]. While the core domains of the ATPase are conserved, the N- and C-terminal regions are variable (Fig 2b). The archaellum-specific ATPase shows the simplest organization, while other subsystem ATPases carry extensions at their N- and/ or C-terminus. Predictions of the hexameric assembly suggest that these ATPases might form hexamers as seen for the archaellum ATPase with the extra domains accessible to other T4F components (S8 Fig) [5]. We hypothesize that these extensions serve as interaction interfaces to subsystem-specific accessory proteins. Consistent with this, functionally characterized ATPases and T4F machineries in Sulfolobales show that distinct functions and mechanisms correlate with the structural variety [5,6,8,11], and cross-complementation of ATPases and platform proteins in *Haloferax volcanii* is hampered, likely due to mismatched extra domains [30].

**Fig 2:**
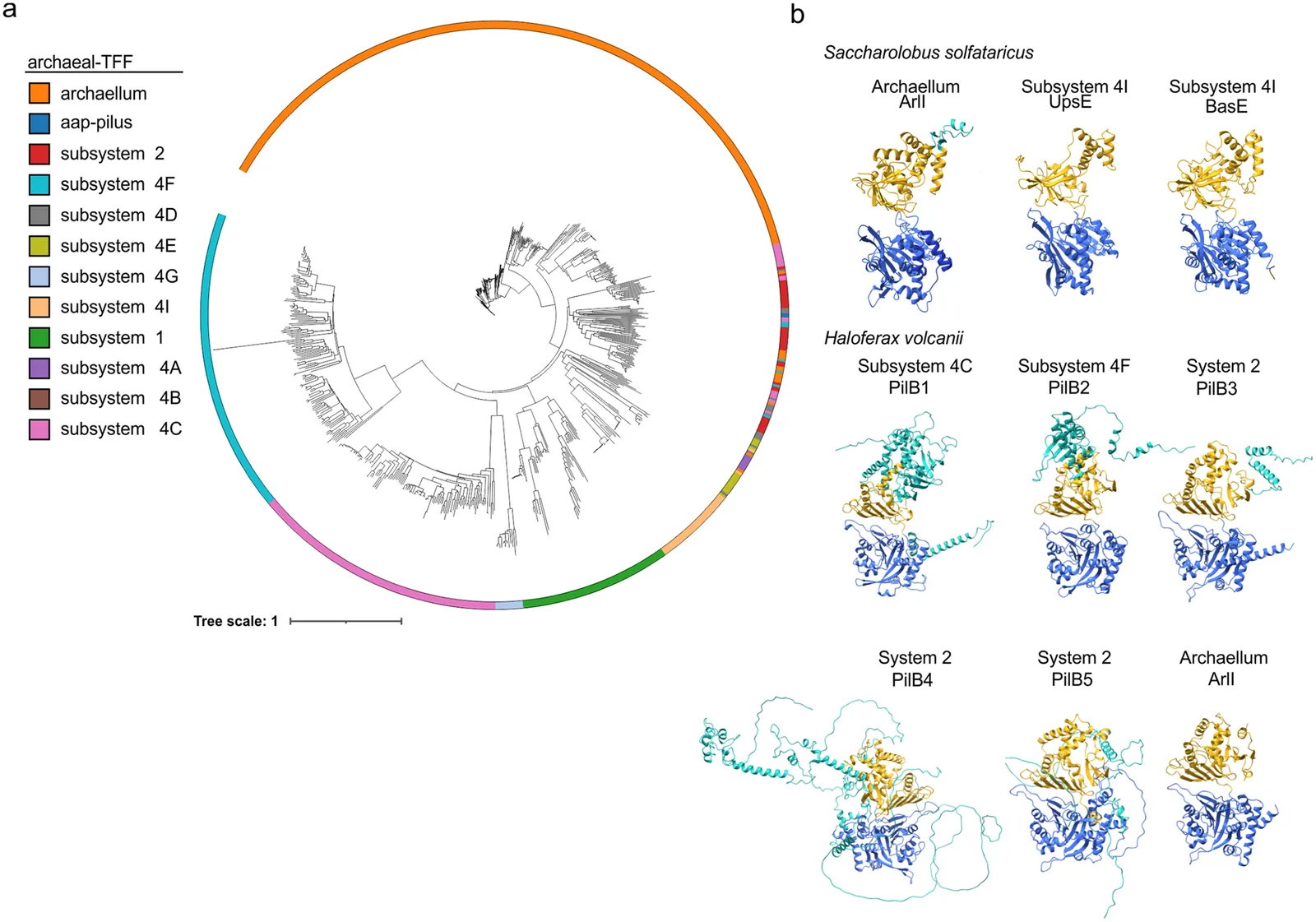
Structural phylogeny of selected T4F related ATPases. **a** Structure-guided phylogenetic tree of ATPases using fold_tree labelled by archaeal T4F system. Tree scale as indicated. b Alphafold predicted structures of T4F-related ATPases from the model organisms *Saccharolobus solfataricus* and *Haloferax volcanii* showing the structural variability across T4F systems. Structures are coloured according to the core and variable domains. The N-terminal region is shown in gold and, while the C-terminal part, coloured in blue, shows the ATP-binding and hydrolysis region. The system-specific extensions at the N- and C-termini are shown in teal.

### The archaellum machinery is widespread in archaea

The archaellum, the motility machinery, is the most widespread T4F identified. 32% of identified replicons are frequently accompanied by additional T4F machineries of other subsystems, suggesting that each T4F system evolved towards a dedicated function that limits cross-compatibility (Fig 1d and 1f). Most archaella share a conserved core of ArlJ and ArlI, the platform and ATPase components, respectively (Fig 3). Key accessory genes within the replicon define the archaellum gene locus. The order of *arlF* and *arlG* together with the presence of either *arlCDE* homologs or *arlX* delineates the archaellum of Thermoproteota and Methanobacteriati [17,31]. Most Thermoproteota (except Nitrososphaerota) encode *arlX*, while methanobacterial species encode *arlCDE* homologs in various fusion or fission arrangements. The presence of *arlCDE* typically coincides with that of the archaea-specific chemotaxis adapter *cheF*, in agreement with experimental evidence that CheF interacts with ArlCDE [32–34] (Fig 3). In addition, homologs of arCOG05057 were found in some Methanococcales, encoding an S-layer-like protein composed of several Ig-like folds (Fig 3). A similar S-layer-like protein in Thermococcales archaellum loci is found, indicating that this association is more widespread than previously thought [17]. The resemblance to known archaeal S-layers suggests a role in priming or locally modifying the S-layer to allow the filament to traverse the otherwise rigid, impermeable cell envelope.

**Fig 3:**
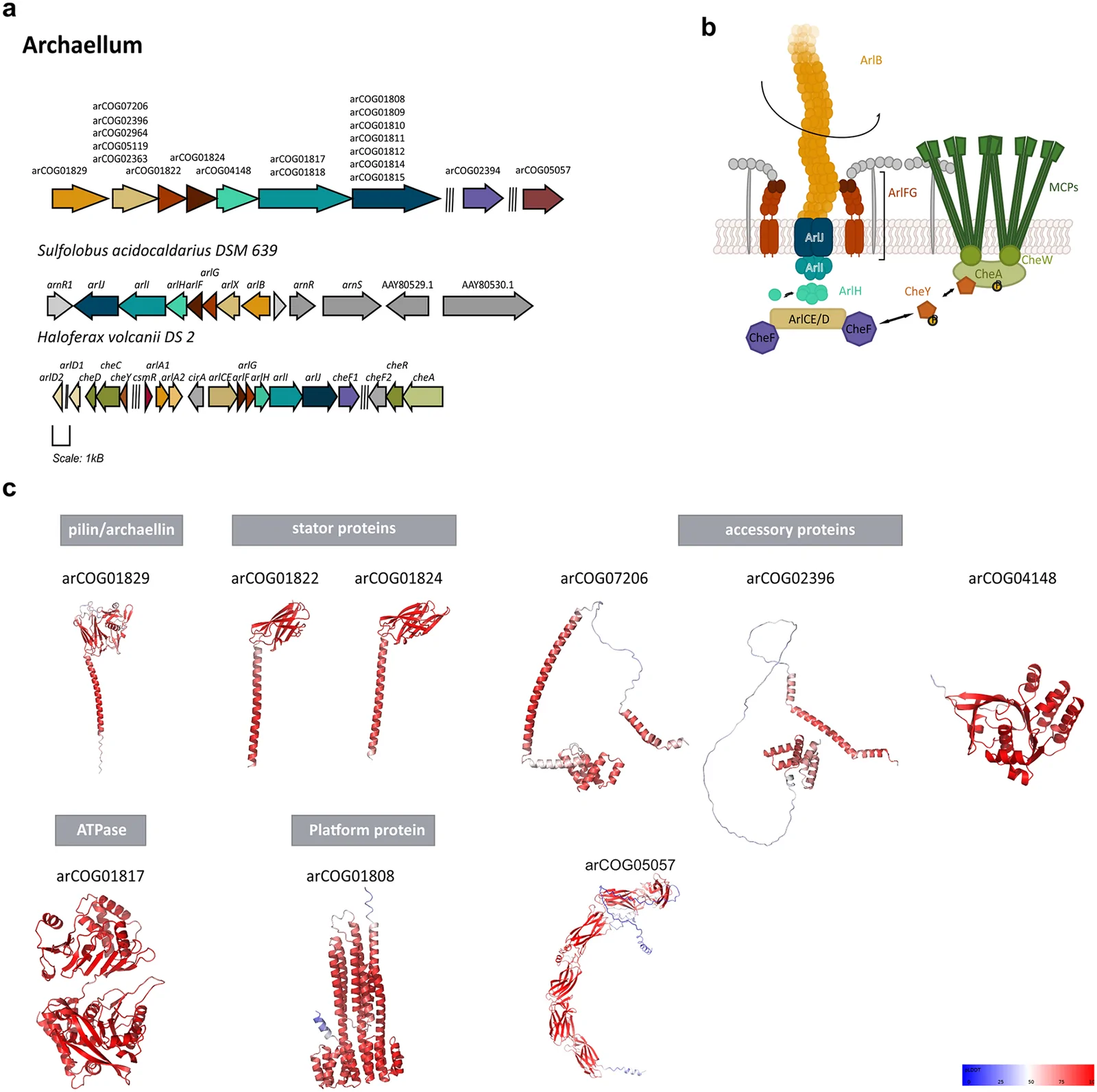
Schematic representation of archaellum T4F. **a** Gene locus organization with corresponding arCOGs in exemplary organisms encoding an archaellum T4F. **b** Schematic of the archaellum machinery and its accessory components. **c** Alphafold database structures of the core archaellum components, coloured by pLDDT value (blue-low, red-high).

### Aap pilus

The Aap pilus is well characterized in Sulfolobales and allows for twitching motility in the absence of a dedicated retraction ATPase [7]. It mediates surface adhesion and biofilm formation [6] and consists of two pilin homologs, an ATPase, a platform protein, and a protein bearing an Fe–S cluster domain (Fig 4). Aap specific pilins belong to arCOG03871 and arCOG03872 (Fig 4a). Structural analysis showed that a single pilin subunit can adopt three conformations, with varying angles between the hydrophobic N-terminal region and the globular domain, providing filament flexibility required for surface-associated motility [35]. The Fe–S cluster protein (arCOG07320) is found in most Sulfolobales and in some Desulfurococcales, Thermoplasmatales and Nitrososphaerota, including *Nitrososphaera koreensis*, *Nitrosocaldus uzonensis*, *Nitrosopumilus maritimus*, and *Ca.* Udinarchaeum marinum, but its function is unknown. Some lineages, such as *Ca.* Jingweiarchaeum tengchongense, have multiple Aap loci. Simpler Aap-like systems lacking the *aapX* homolog occur in several Heimdallarchaeota, Bathyarchaeota, and Nanobdellati members. These minimal systems may still be functional: *Ignisphaera aggregans*, which encodes a reduced Aap-like machinery, displays surface filaments [36]. Aap-like pili, which likely mediate surface adhesion and possibly twitching motility are probably more widespread across Archaea than previously recognized.

**Fig 4:**
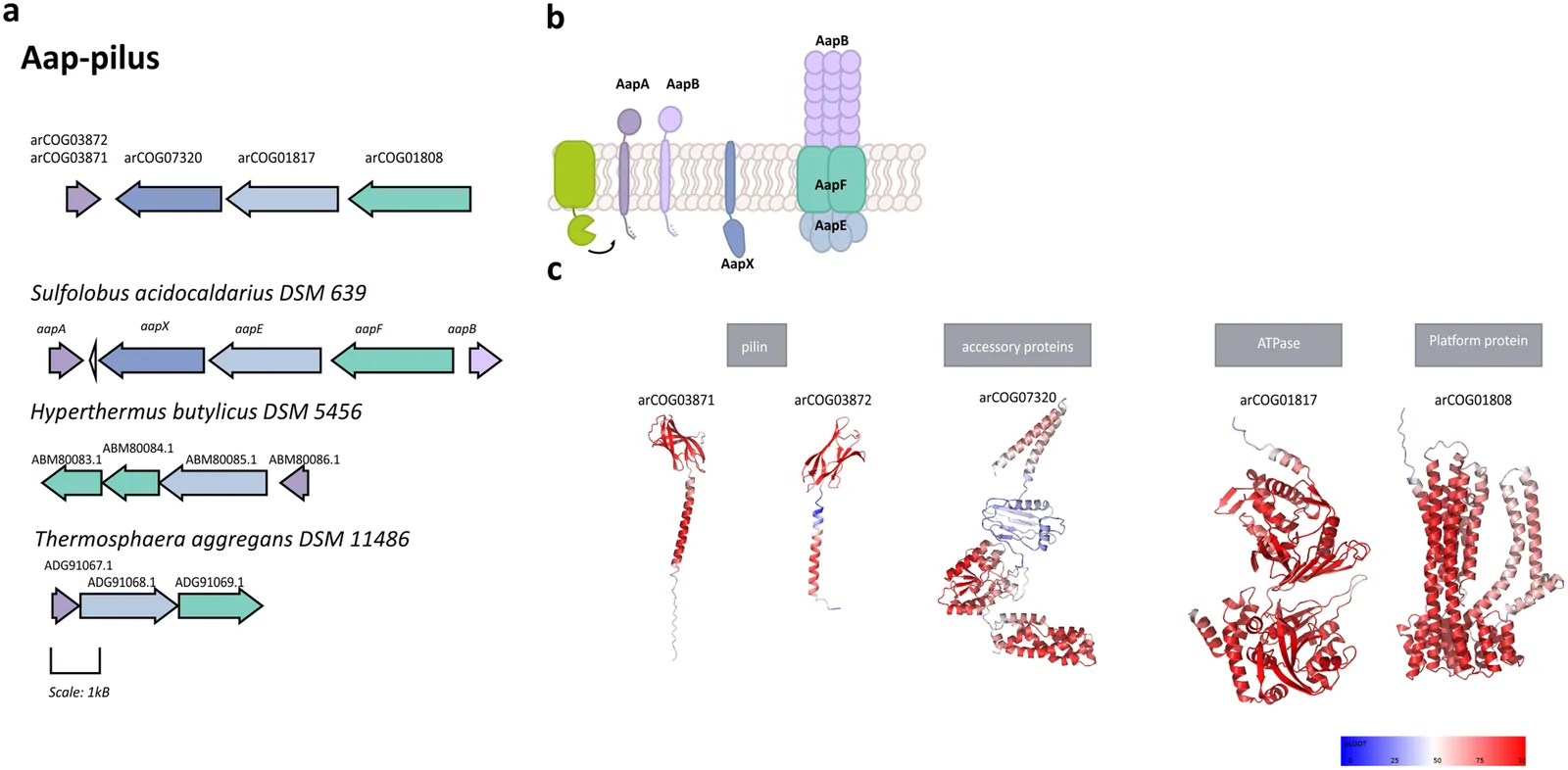
Schematic representation of Aap pilus T4F. **a** Gene locus organization with corresponding arCOGs in exemplary organisms encoding an Aap T4F. **b** Schematic of the Aap machinery and its accessory components. **c** Alphafold database structures of the Aap components, coloured by pLDDT value (blue-low, red-high).

### System 1 is mainly found in methanogens, Nanobdellati and deep-branching Methanobacteriati

Archaeal T4F system 1 clusters are primarily found in Methanococcales, Methanobacteriales, Thermococcales and Nanobdellati. The system includes an ATPase homolog and is characterized by two genes encoding the platform protein (S1 Fig), together with a high variety of pilins and several accessory proteins. This diversity may support two system 1 types: the Epd pilus in Methanococcales such as *Methanococcus maripaludis* S2, and an unidentified pilus composed of the remaining pilins. Indeed, *Haloferax volcanii* encodes six pilin homologs, all expressed and each likely forming distinct filaments [37]. The primary Epd-specific pilin (arCOG06620) lacks the C-terminal globular domain, similar to the bacterial Tad pilus, indicating that the bacterial Tad pilus originated from the archaeal Epd pilus [1]. Additional pilins are encoded in a separate gene locus likely associated with the Epd pilus, as shown for *M. maripaludis* [38,39]. Previously unrecognized system 1 pili in *Methanobrevibacter* species may aid adhesion within the gut microbiome or even host gut epithelia. *Pyrococcus furiosus* and other Thermococcales encode an Epd pilus, underscoring the wide distribution of system 1 T4F in archaea (S1 Fig). The Epd pilus contributes to adhesion. Its 11-gene cluster has been extensively studied and is constitutively expressed as a single transcriptional unit with its own dedicated signal peptidase, EpdA (arCOG2300) [40]. Deleting any of the 11 genes abolishes pilus formation, whereas removing minor pilins markedly reduces pilus formation [13,38]. An adhesin-like domain in Mmp0235 and a SPIII protein with a galactose-binding domain suggest roles in surface recognition and adhesion.

### System 2

Archaeal T4Fs of system 2 are predominantly found in Methanobacteriati (S2 Fig). Often, multiple loci coexist in a single genome. In Halobacteriales, two T4F loci are present, but only the *pil1* locus has been shown to produce functional pili [41]. Similarly, in *Haloferax volcanii*, only *pilBC3* locus forms visible pili that function in adhesion and biofilm formation [28,30,42,43]. The role of the remaining loci is unclear, as similar loci are found in *Aciduliprofundum boonei* and *Archaeoglobus fulgidus*. We show that multiple T4F of system 2 loci coexist in a single genome, suggesting distinct or condition-specific functions. Makarova et al. proposed that system 2 T4Fs may depend on MinD-like ATPases [17]. Indeed, MinD ATPases are widespread and essential for positioning the archaellum in *H. volcanii* [44], pointing to a subsystem-specific regulation analogous to that of the archaellum. System 2 T4Fs differ from system 1 by having ATPases with extended N-terminal and sometimes additional C-terminal regions. The platform protein is encoded by a single gene rather than two. They encode multiple pilins, including SPIII-type and pilin-like proteins without SPIII motif, alongside membrane proteins likely part of the assembly machinery. System 2 loci often encode large adhesin-like proteins that may function in cell adhesion and recognition (S2 Fig). Comparative analysis of organisms with system 2 loci will be needed to determine whether additional surface structures co-occur and whether the encoded genes are expressed under certain conditions.

### T4F clusters belonging to System 4

The clusters assigned to system 4 are highly diverse and can be divided into several subsystems (4A–4I) that differ substantially in gene neighborhood around the platform protein and ATPase [17]. The order and domain architecture vary markedly across subsystems, suggesting frequent gene rearrangements and acquisition of accessory domains during evolution. Each subsystem is described in more detail:

### Subsystem 4A and 4B

Subsystem 4A is predominantly found in Thermoproteota, some Nanoarchaeota, Bathyarchaeota, and a few genomes of Methanobacteriati. The cluster organization is distinct, with the accessory components found downstream of the platform protein (S3a-S3c Fig). The minimal subsystem 4A has two platform proteins, an ATPase, a pilin, and often an adhesin (S3 Fig). Its pilins belong to arCOG11238 and arCOG08836, including a subsystem 4AB specific adhesin (arCOG03511) that is remotely homologous to bacterial PilY. In bacteria, PilY is thought to contribute to adhesion and mechanosensing, linking type IV pili dynamics to intracellular regulation via cyclic nucleotide levels [45,46]. In *Pseudomonas aeruginosa*, PilY forms a corkscrew-shaped structure at the secretin, gating filament assembly and regulating the cycles of extension and retraction [45].

Subsystem 4B has a relatively simple organization comprising two pilins with genes encoding the platform protein and an ATPase. Two platform encoding genes are flanking the ATPase gene, which is different than in other T4F loci (S3d-S3f Fig). This subclade contains arCOG07037 near the major pilin gene, encoding an adhesin-like protein that strongly resembles bacterial hook proteins built from several Ig-fold domains, suggesting a role in cell attachment (S3f Fig). Subsystem 4B loci are restricted to a few lineages, including *Sulfolobus islandicus*, S*accharolobus caldissimus*, *Thermoproteus* neutrophilus, and *Thermoproteus uzoniensis*. In *Thermoproteus tenax*, the 4B clusters are associated with RecA-like ATPase, cystathionine-β-synthase (CBS) domain proteins, and a eukaryotic DEATH domain protein, which seems to replace KaiB found in some 4B loci [17]. This suggests functional coupling between T4F assembly and cellular regulatory networks as seen for the complex regulation of the archaellum (reviewed in [47]).

### Subsystems 4C and 4D

The 4C subsystems are found predominantly in halophilic archaea, often co-occurring with subsystems 4F and 4G. Each encodes a platform protein and an ATPase decorated with accessory pilins and membrane-inserted proteins. Distinctive extra loops at the N and C-termini of the platform protein and ATPase may be crucial for the function of the archaeal T4P systems. Associated pilins vary in their head domain, which could reflect specialized functions such as cell adhesion or recognition (S4a- S4c Fig). Like subsystem 2, subsystem 4C seems specific to halophilic members of Methanobacteriati but forms its own subsystem due to the gene arrangement and distinct pilins and accessory proteins. Makarova et al. propose a hybrid origin for this system, with membrane machinery resembling thermoproteal members and pilins likely of methanobacterial origin [17], implying that combining base machinery from different members can yield functional systems if core components are compatible. In contrast, subsystem 4D is notably distinct, comprising an ATPase, a platform protein, but no putative pilin. The presence of a TFIIB-like transcription factor specific to *Pyrobaculum* and *Thermoproteus*, as well as an RNA polymerase, suggests that it may be a nonfunctional remnant of a T4F system that was repurposed for a different function (S4d -S4f Fig) [17].

### Subsystems 4E and 4F

Members of Thermoproteota specifically from *Pyrobaculum* and *Vulcanisaeta*, encode a subsystem 4E pilus machinery. Its core is likely linked to arCOG03739 and arCOG03740 pilins near the T4F locus (S5a – S5c Fig) [17]. The locus is split into membrane-embedded machinery genes and distantly located pilin genes (S5a-S5c Fig). Functional expression of an assembled filament was shown for *P. arsenaticum 2GA* which forms long thin filaments that serve as attachments for archaeal viruses [48]. The pilin carries an extra C-terminal domain and cysteines that likely enhance stability in hyperthermophilic environments. Subsystem 4F resembles subsystem 4C in gene arrangement, distribution and its the hybrid character of core machinery and accessory components [17]. The 4F locus co-localizes with the archaeal tubulin homolog CetZ2, a cystathionine-β-synthase (CBS) domain protein, or a structural maintenance of chromosomes (SMC) homolog (S5d – S5f Fig) and contains an adhesin-like protein (arCOG02945). These genes may be important for positioning and regulating the components of subsystem 4F.

### Subsystems 4G and 4I

Subsystem 4G is highly complex, encoding several pilins, membrane inserted accessory proteins and all systems co-occur with a MinD-like ATPase (S6 Fig). It is found mainly in Pyrobaculum and Thermoproteus, often together with a subsystem 4E T4F locus. Besides its many T4F associated components, they include a large adhesin-like proteins (arCOG05616). This protein, consisting of several Ig folds, resembles the prokaryotic hook-like proteins recently characterized in *Aureispira sp.,* also found in some Chloroflexota [49,50]. In *Aureispira sp.,* such hook-like proteins mediate interaction with prey flagella, enabling ixotrophic behavior. Their function in archaea is unknown but, as in bacteria, they may be involved in adhesion and/or cell-cell recognition. In gut methanogens, adhesins are crucial for the interaction between secreted membrane vesicles and host gut epithelial cells [51].

Subsystem 4I contains the bindosome and Ups pili of Sulfolobales exclusively. Both systems were experimentally shown to be expressed in this group [8,21,52–54]. The Ups pilus is encoded in a locus containing two pilins, an unknown protein (UpsX, arCOG05986) and its platform protein and ATPase (S7a and S7b Fig). It is induced by UV stress and mediates species specific cell-cell interactions for Ced-mediated DNA exchange and DNA repair by homologous recombination [8,12]. Owing to its function, the gene neighborhood is highly conserved and contains a helicase, endonuclease, and a glycosyltransferase that may participate in DNA exchange [16]. In contrast, the bindosome is characterized by three pilins located close to the membrane spanning assembly machinery encoded by *basEF* [54] (S7d and S7e Fig).

Several Sulfolobales members use sugar-scavenging bindosome machinery. These bindosomes are associated with an ABC transporter and a substrate binding protein whose N-terminus follows the T4F pilin blueprint [21,54,55]. While the core machinery is found in one gene locus, the sugar binding proteins are encoded mostly within the ABC transporter locus. These sugar binding proteins are processed by the class III signal peptidase. After processing these low-affinity sugar binding proteins are inserted into the bindosome [55]. The base machinery and binding proteins are regulated differently and adapt to substrate availability as the binding proteins are induced by the presence of specific sugars [56]. Deletion of the bindosome substantially reduces glucose uptake via its specific binding protein, indicating that T4F base machinery, BasEF in Sulfolobales serves the sugar binding proteins GlcS, AraS, and TreS, however, growth is not delayed [20,21,56].

### Widespread presence of bindosomes with a broad substrate range in archaea

Class III signal peptide proteins encompass both T4F-related pilins and substrate-binding proteins, the latter predominantly specific for sugars. In Sulfolobales such as *Saccharolobus solfataricus*, around 8 sugar-binding proteins with a class III signal peptides have been identified [40]. These proteins share a conserved N-terminus but diverse C-terminal regions due to their binding properties. They often co-localize with ABC transporters rather than T4F clusters, indicating regulation by substrate availability. Despite having 8 binding proteins, *S. solfataricus* has only one bindosome T4F machinery that likely serves multiple binding proteins depending on the available substrate. To elucidate whether more archaea functionalize a T4F for substrate binding, archaeal genomes were screened for proteins with a class III signal peptide and the candidates with potential binding function were analyzed (Fig 5). Possible signal peptides with a lipobox motif indicating signal peptidase II and Tat substrates were excluded, leaving 4397 potential binding proteins with a class III signal peptide (Fig 5a). Notably, these proteins not only reside in Sulfolobales but also in several methanobacterial members. Most genomes contained one or two such proteins, although up to nine were also found (Fig 5c). About 3021 binding proteins occurred in genomes that also encoded a T4F machinery that could serve as the base, whereas 1376 genomes with an SPIII binding protein lacked a T4F locus. Of these, 801 also contained a putative SPIII peptidase that would allow correct processing of the binding protein (Fig 5a).

**Fig 5:**
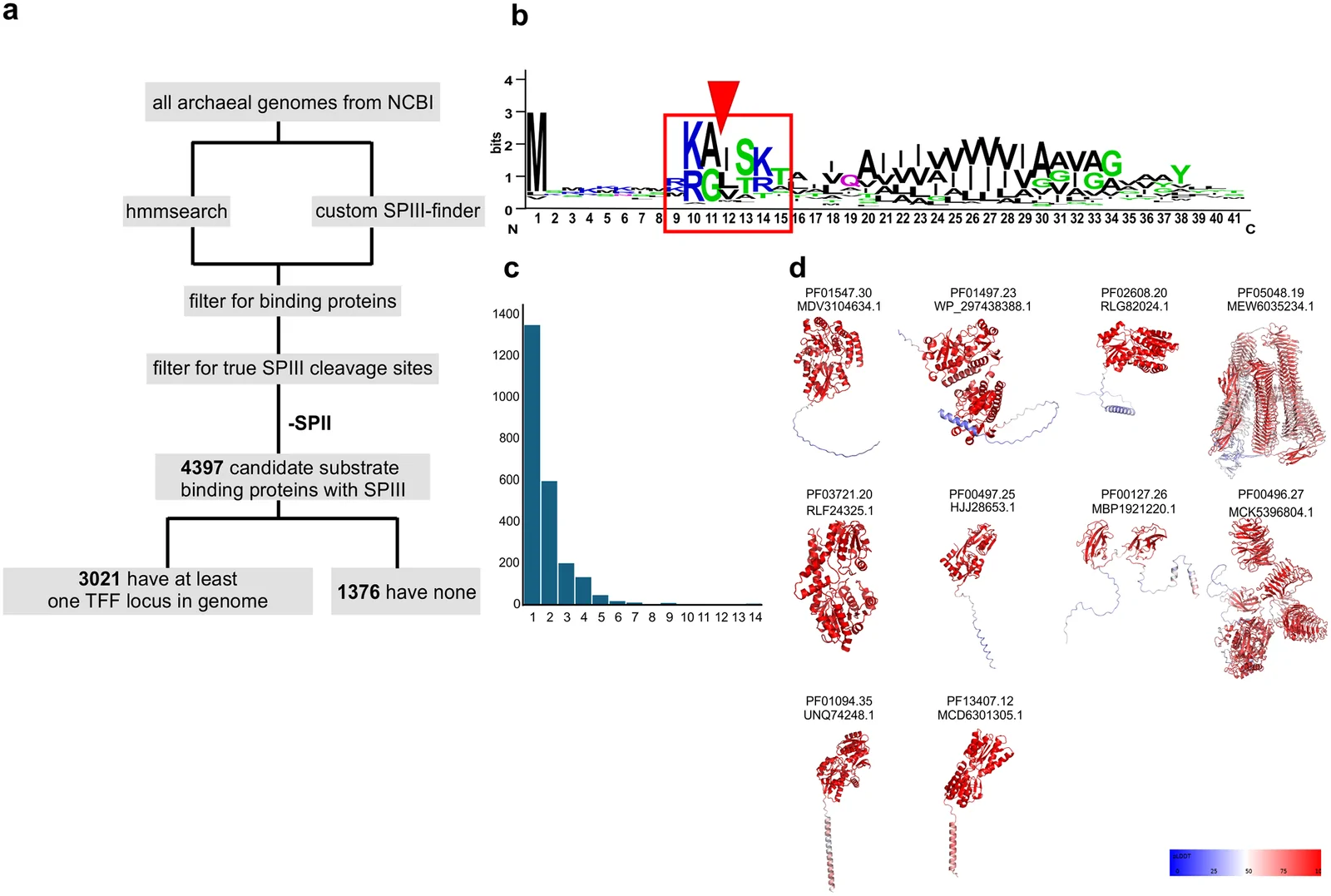
Identification of putative binding proteins with a SPIII signal peptide. **a** Schematic of the identification of binding proteins. **b** Weblogo of N-termini of identified binding proteins with conserved SPIII cleavage site and hydrophobic domain as indicated. **c** Frequency of identified binding proteins per genome. **d** AlphaFold3-predicted structure of top 10 PFAM domains showing variable c-terminal organization and a hydrophobic N-terminal helix. Structures are coloured by pLDDT value (blue-low, red-high).

We mapped the PFAM domains identified by hmmscan to putative binding proteins. The top ten candidates are almost all involved in substrate binding, which is commonly associated with ABC transporters (S9 Fig). The exact substrate is unknown for most, but the binding range likely spans from sugars, peptides to specific molecules and metal ions. Structural predictions of representative binding proteins per PFAM reveal diverse C-terminal architectures, from short globular folds to long β-helical structures (Figs 5d, S9). All share a hydrophobic TM-domain preceding the functional domain and an SPIII cleavage site as seen in pilins and archaellins. It is tempting to speculate a link between SPIII-containing substrate binding proteins and T4F locus which would facilitate substrate scavenging in archaea. An alignment of all extracted N-termini showed highly conserved positive charges preceding the hydrophobic domain, consistent with the well-studied class III signal peptide (Fig 5b). However further experimental validation is needed to correlate the presence of substrate binding proteins to specific T4F gene locus.

## Discussion

### Widespread diversity of archaeal T4F machineries

A systematic screening of archaeal type IV filament (T4F) identified 29,521 T4F-related loci and many archaeal genomes with multiple T4F systems, which displayed a remarkable diversity. All systems share the essential components defining a T4F machinery: a single ATPase, a membrane platform protein, a class III signal peptidase, and at least one filament forming pilin (Fig 1C). Each subsystem adds accessory proteins that are unique and critical to its respective functions. While multiple pilin and platform protein genes are found, the ATPase gene remains in a single copy per subsystem, with no evidence of gene fission or fusion, in contrast to bacterial T4F systems where ATPase duplication has occurred for different functions [1]. Archaeal pilins, both major and minor, are located within subsystem loci or as standalone genes. This suggests that the current model used by MacSyFinder2 may not capture the full pilin repertoire. Whereas the ordered search identified T4F loci within a single genomic locus, the unordered search revealed candidate pilins and T4F machinery scattered across genomes in Prometheoarchaeati and Nanobdellati (S10 Fig). It remains unclear whether these T4F genes actually produce functional surface structures. Nonetheless, it is puzzling that two phyla harbor an unordered T4F gene organization from the otherwise ordered replicon organization seen across most archaeal phyla. It is unclear whether these components were acquired independently or whether genome expansion in Prometheoarchaeati and genome reduction in Nanobdellati split apart an originally contiguous cluster.

Most genomes frequently contain more than one T4F locus, with its specific set of pilins and structurally diverse core machinery (Fig 2) that points to specialization rather than redundancy. In *Haloferax volcanii*, cross-complementation experiments showed that base machinery of one T4F locus cannot rescue deletion phenotypes in another, indicating functional divergence [30]. Comparative analysis of ATPase and platform proteins in genomes with multiple systems, such as *H. volcanii* and *S. solfataricus*, reveals significant structural differences, particularly in the N- and C-terminal domains of ATPases, which further limits cross-complementation (Fig 2). Structure-guided phylogenetics show that ATPases group according to their T4F system, reflecting structural diversification driven by adaptation to distinct cellular functions. Beyond the core ATPase, platform protein (ArlJ family), and class III signal peptidase, we found no additional genes conserved across T4F loci that are not already described by the existing arCOGs, which is consistent with the core set defined by Denise et al. (2019). Many subsystems include proteins with multiple Ig-folds, adhesin-like domains, or β-helical sheets. Subsystem 4A/B contains a PilY-like adhesin implicated in cell adhesion and surface recognition. The resemblance of some to archaeal S-layer proteins suggests a role in priming the cell wall so that the filament can traverse through the envelope. Notably, modeling of S-layer and surface filaments of *S. acidocaldarius* indicates that local S-layer rearrangements are required for pilus insertion [57].

### Functionalized T4F machineries for substrate binding and nutrient scavenging

Substrate-binding proteins with class III signal peptides (SPIII) are widely distributed. They have been firstly identified in Sulfolobales but also found in other archaeal lineages [40] (Fig 5). They share highly conserved N-termini with class III signal peptide and a hydrophobic α-helix that may mediate integration into a pilus structure. Alpha-helical interactions stabilize the protein within the filament and contribute to its architecture. In bacteria, pilus filaments are rarely built from a single pilin. Minor pilins and tip complexes drive functional diversification such as DNA uptake and adhesion for cell flocculation [58–60]. These observations support the hypothesis that a base T4F machinery may serve as a versatile platform for multiple substrate-binding proteins for efficient substrate binding and uptake via the associated ABC transporter. In *S. solfataricus*, sugar-binding proteins are induced by their sugar availability and they rely on the bindosome base structure [20, 21, 56]. Indeed, surface proteome studies in *Aeropyrum pernix* detected a substrate binding protein with a class III signal peptide, indicating assembly outside Sulfolobales [61]. The ability of T4F subsystems to accommodate various substrate-binding proteins points to a functional range extending well beyond motility, adhesion and sugar uptake. This diversity is seen in the structural variability of the N-terminal ATPase domain which likely arose through extensive evolutionary diversification and incorporation of novel, repurposed components (Fig 5). While the core ATPase and platform protein retain structural similarity, additional domains which have evolved in each subsystem limit cross-complementation and point to specialization for distinct functions. Archaea occupy a wide range of environments and the variability in T4F systems reflects this diversity. Future studies combining genetics, structural biology, and *in vivo* localization will be essential to fully define the novel functions of T4F machineries.

## Materials and methods

### Identification of T4F machineries

Archaeal Type IV filament systems were identified in archaeal genomes with MacSyFinder2 [18]. A total of 16,694 archaeal genomes (NCBI, May 2025) were screened using custom MacSyFinder models designed to capture the diversity of archaeal T4F subsystems [18]. Models were built for each T4F subsystem following Makarova et al., 2016 [17]. Briefly, Hidden Markov model (HMM) profiles for T4F-associated arCOGs were retrieved from eggnog [62] and sorted by subsystem based on their functional annotation. For each model, the ATPase, platform protein, and prepilin peptidase were defined as mandatory components (S2 Table), with the remaining pilins and subsystem-specific accessory proteins defined as accessory (S3 Table). Mandatory genes were additionally linked through exchangeable-gene sets to account for paralog diversification within each component accounting for different arCOGs per ATPase, platform protein and signal peptidase. All models required a minimum of 2 mandatory genes and 3 total genes per system (4 for *archaellum*), allowed multiple loci per genome (multi_loci = 1), and used a maximum inter-gene spacing of 5 genes to define co-localized clusters (inter_gene_max_space = 5). The signal peptidase was set as loner outside the co-localized locus and to be shared across multiple systems MacSyFinder (v2.1.6) was run with --no-cut-ga and an e-value cutoff of 1e-3 and profile coverage cutoff of 0.3 for hit selection. Genomes were screened in ordered_replicon mode using a custom wrapper script that parallelized MacSyFinder runs across genomes, requiring co-localization of mandatory and accessory genes within a single locus for robust subsystem assignment. The presence of accessory genes in gene neighborhood defined which subsystem they belong to prometheoarchaeal genomes and Nanobdellati were specifically screened in unordered replicon mode to detect candidate T4F-related genes due to fragmented T4F locus.

### Analysis of gene locus

The exemplary gene locus per system from MacSyFinder2 output was visualized with Gene Graphics (Harrison et al., 2018).

### Structure guided phylogenetic tree

ATPases of high confidence T4F clusters were extracted and uniprot identifiers and corresponding Alphafold predicted structures were retrieved using a custom python script written with help of the GPT-4.1 AI model. Fold_tree 1.0.0 [19] was used to build a structure guided phylogenetic tree with the command snakemake --cores 24 --use-conda -s  ./workflow/fold_tree --config folder = ./myfam filter = False custom_structs = True.

### Mapping archaeal T4Fs onto a reference phylogenetic tree

A reference phylogenetic tree of archaeal genomes was derived from the GTDB (release 10, May 2025) [22]. The detected T4F systems were mapped onto this tree to visualize their distribution in archaeal lineages, using colour-coded annotations for each subsystem and the number of T4F loci per genome [63].

### Identification of binding proteins with SPIII

SPIII-containing proteins were identified with a custom archaeal signal peptide script written with the help of the GPT-4.1 AI model. This script combines detection of N-terminal transmembrane helices (TMHMM 2.0) with SPIII specific cleavage motif according to Szabo et al., 2007 [40], while also screening for Tat secretion signals and lipoboxes. Proteins with a TM segment within the first 30 amino acids and a SPIII cleavage site were retained. HMMER v3.4 (hmmsearch) was used to identify conserved domains in these proteins using PFAM database [64]. Predicted structures were retrieved from the Alphafold database and rendered in PyMOL.

### AI tool statement

During the preparation of this manuscript, ChatGPT was used to correct the English language. After using this tool/service, the chapter was reviewed, and the content was edited as needed, taking full responsibility for the content of the publication.

## Supporting information

S1 Fig Schematic representation of System 1. **a** Gene locus organization with corresponding arCOGs in exemplary organisms encoding a system 1 T4F. **b** Schematic representation of the system machinery with its accessory components. **c** Alphafold database derived predicted structures of system 1 related components. Structures are coloured according to pLDDT value (blue-low, red-high).
(TIF)

S2 Fig Schematic representation of System 2. **a** Gene locus organization with corresponding arCOGs in exemplary organisms encoding a system 2 T4F. **b** Schematic representation of the system 2 machinery with its accessory components. **c** Alphafold database derived predicted structures of system 2 related components. Structures are coloured according to pLDDT value (blue-low, red-high).
(TIF)

S3 Fig Schematic representation of subsystem 4a and b. **a,d** Gene locus organization with corresponding arCOGs in exemplary organisms encoding a system 4a and 4b T4F. **b,e** Schematic representation of the system 4a and 4b machinery with its accessory components. **c,f** Alphafold database derived predicted structures of system 4a and 4b related components. Structures are coloured according to pLDDT value (blue-low, red-high).
(TIF)

S4 Fig Schematic representation of subsystem 4c and d. **a,d** Gene locus organization with corresponding arCOGs in exemplary organisms encoding a system 4c and 4d T4F. **b,e** Schematic representation of the system 4c and 4d machinery with its accessory components. **c,f** Alphafold database derived predicted structures of system 4c and 4d related components. Structures are coloured according to pLDDT value (blue-low, red-high).
(TIF)

S5 Fig Schematic representation of subsystem 4e and f. **a,d** Gene locus organization with corresponding arCOGs in exemplary organisms encoding a system 4e and 4f T4F. **b,e** Schematic representation of the system 4e and 4f machinery with its accessory components. **c,f** Alphafold database derived predicted structures of system 4e and 4f related components. Structures are coloured according to pLDDT value (blue-low, red-high).
(TIF)

S6 Fig Schematic representation of subsystem 4G. **a** Gene locus organization with corresponding arCOGs in exemplary organisms encoding a system 4g T4F. **b** Schematic representation of the system 4g machinery with its accessory components. **c** Alphafold database derived predicted structures of system 4G related components. Structures are coloured according to pLDDT value (blue-low, red-high).
(TIF)

S7 Fig Schematic representation of subsystem 4I. **a,d** Gene locus organization with corresponding arCOGs in exemplary organisms encoding a system 4i UV pilus and bindosome T4F. **b** Schematic representation of the system 4i machinery with its accessory components. **c,e** Alphafold database derived predicted structures of system 4i related components. Structures are coloured according to pLDDT value (blue-low, red-high).
(TIF)

S8 Fig AlphaFold3 predictions of ATPase hexamers in *Saccharolobus solfataricus* and *Haloferax volcanii.* Hexameric structure predictions of ATPases from the archaellum and T4F subsystems in *Saccharolobus solfataricus* (top) and *Haloferax volcanii* (bottom), shown in top view and side view. Each structure is colored in rainbow from N-terminus (blue) to C-terminus (red).
(TIF)

S9 Fig All PFAM domain identified from hmmsearch of binding proteins shown in Fig 5. (TIF)

S10 Fig Gene loci of identified components of a potential T4F system in representative (a) asgardarchaeal and (b) DPANN archaeal genomes. (TIF)

S1 Table Overview of archaeal T4F subsystems. Taxonomic distribution, conserved core components, subsystem-specific components, distinguishing features and key references for each subsystem.
(XLSX)

S2 Table Mandatory components of the MacSyFinder2 models. arCOGs defining the ATPase, platform protein and class III signal peptidase. (XLSX)

S3 Table Subsystem-specific accessory arCOGs. Protein names, annotation (after Makarova et al. [17]) and number of organisms detected with MacSyFinder2.
(XLSX)

S4 Table T4F-related genes detected in Prometheoarchaeati and Nanobdellati genomes by the unordered search. (XLSX)

S5 Table Subsystem assignment of unordered T4F loci in Prometheoarchaeati and Nanobdellati. (XLSX)
