# Supplementary material for: Diversification and functional expansion of archaeal T4F machineries
Source: PLoS Genet. 2026 Oct 14;22(10):e1012139. doi: 10.1371/journal.pgen.1012139 (PMC13705601; doi:10.1371/journal.pgen.1012139)
Supplement: Supplementary file 1 [file pgen.1012139.s017.docx]

**Reviewer answers for the submitted manuscript “Diversification and expansion of archaeal T4F machineries**

**Additional Editor Comments:**

Your manuscript has been carefully read by three reviewers. The reviewers note that overall the manuscript is well organized and represents a useful update to the classification of archaeal TFF systems. However, they have raised a number of concerns and have offered many suggestions for improvement. In particular, reviewers 1 and 3 raised important concerns about speculative over-interpretation of the data presented. Please carefully consider what conclusions can be drawn from the data and tone down functional speculations that lack supporting experimental evidence.

In addition, all of the reviewers raised concerns about the details of the presentation, both in terms of the writing and the presentation of figures. The reviewers have provided many specific examples in the text that need to be corrected or clarified. Please carefully edit a revised manuscript.


**Journal Requirements:**

1) Please ensure that the CRediT author contributions listed for every co-author are completed accurately and in full.
At this stage, the following Authors/Authors require contributions: Shamphavi Sivabalasarma, and Sonja Albers. Please ensure that the full contributions of each author are acknowledged in the "Add/Edit/Remove Authors" section of our submission form.
The list of CRediT author contributions may be found here: <https://journals.plos.org/plosgenetics/s/authorship#loc-author-contributions>

2) We have noticed that you have uploaded Supporting Information files, but you have not included a list of legends. Please add a full list of legends for your Supporting Information files after the references list.

3) Please amend your detailed Financial Disclosure statement. This is published with the article. It must therefore be completed in full sentences and contain the exact wording you wish to be published.
State what role the funders took in the study. If the funders had no role in your study, please state: "The funders had no role in study design, data collection and analysis, decision to publish, or preparation of the manuscript.".
If you did not receive any funding for this study, please simply state: u201cThe authors received no specific funding for this work.u201d

**Reviewers' comments:**

Reviewer's Responses to Questions

**Comments to the Authors:
Please note here if the review is uploaded as an attachment.**

Reviewer #1: General comments
The article by Sivabalasarma and Albers describes an evolutionary and functional classification of archaeal type IV filament (T4F) nanomachines. This work is an update of an original classification established in 2016 by Makarova, Koonin and Albers (doi: 10.3389/fmicb.2016.00667) which was based on 168 archaeal genome sequences. Based on the sequence analysis of their ATPase motors, the T4F systems were grouped into 4 main clades, with clade 4 branching into 10 different subgroups. In the present manuscript, the authors dispose of a larger sequence database from over 16500 archaeal genomes and use an updated version of the search tool - MacSyFinder2. They added AlphaFold-generated structural predictions on T4F components and construct a structure-based tree of T4F ATPases. The overall system classification remains similar to the previous one with 12 T4F system groups instead of 13 by Makarova et al. They propose that the retractable aap pilus be considered as a separate group, while the previous subgroups 4H and 4J disappear from the classification. The authors demonstrate the diversification of ATPases, with members of some groups acquiring new domains at their extremities. However, structural predictions of these domains are poor and their function remains unknown. The authors also highlight the presence of substrate binding proteins with class III signal peptides, which are widespread presumably beyond the characterised Sulfolobus bindosome systems of group 4I. However, it was not clear whether these binding proteins are connected to different T4F groups and if so which ones.

*A: We thank the reviewer for the exhaustive and detailed comments on the manuscript.*

Overall, despite the availability of a larger database, there are few novel findings and ideas that emerge from this analysis. There is also little discussion on potential evolutionary scenarios. It would have been interesting to discuss the influence of other cellular factors, notably of the cell envelope structure and composition, on the evolution of T4F systems.

Even though the authors used AI tools during writing, the text lacks precision and clarity and is filled with errors. Inconstancies in use of terminology between genes, proteins, COGs are found throughout. A gene does not encode for a protein but either encodes a protein or codes for a protein. Sentences like “the platform protein is a single gene” (l. 258) or “pilins belong to arCOG…” (l.281) are not acceptable for a genetics journal.

*A: We thank the reviewer for pointing out inconsistencies. We revised the manuscript carefully and tried to remove these.*

Below I provide probably a non-exhaustive list of specific comments:
l.13: Abbreviation TFF is used for type IV filaments. I suggest that T4F would be a little more informative.

*A: We agree with the reviewer that T4F is the more obvious choice of abbreviation and changed it accordingly in the manuscript.*

l.53. Please provide a reference for this statement about filament subunit gene duplication.

*A: Now L. 65. We added references to the statement about filament subunit duplication.*

l.56 remove the word “is” at the end of the line.
l.58. a circular statement, to be modified
l.61. as above, change to e.g. “while all machineries share similar core components, specific accessory components are found in each system”

*A: The typos were corrected, and the above-mentioned sentences were rephrased.*

l.64. Makarova et al. describe 13 subsystems, please explain why 11 are mentioned here.

*A:. Indeed, Makarova et al. described 13 subsystems, though the latter two are not further described in their paper. As there is no distinguishing difference between subsystems 4J and 4H and subsystem 4A, they have been reclassified as subsystem 4A.*

l.67. “….identified several archaeal organisms encoding at least one archaeal TFF system.” Besides having the second “archaeal” word in excess, this sentence is an understatement, as thousands of archaeal genomes have at least one T4F gene cluster.

*A: We agree with the reviewer and rephrased the sentence accordingly, as follows: L66-67: Here, we expanded this analysis on the currently available vast number of archaeal genomes and identified the widespread presence of a T4F-locus.*

L. 70. The reference 17 for MacSyFinder should be moved to the end of the previous sentence in line 69.

*A: The reference was moved to the end of the sentence.*

l.75. Please specify which novel archaeal surface structures were identified here.

*A: We want to specify that the identified surface structures are based on the systems characterized by Kira Makarova. The novelty lies in expanding the analysis to the vast number of archaeal genomes available today. While this does not include novelty in the surface structure systems, we removed the word ‘novel’ and rephrased the sentence.*

l.92. remove “which”.
l.93. the arCOG is an abbreviation for “clusters of orthologous genes”. Please correct.

*A: Both were corrected.*
l. 93-95. please clarify this sentence.

*A: The sentence was rephrased for clarity.*
L.103 please remove “in their genomes”.

*A: It was removed.*
l. 104. Figure 1c is not appropriately referenced in the text.

*A: We referred to Figure1C in the introduction to point towards the core conserved components of the T4F.*
l.105. replace “encode for” by “encode” here and throughout.

*A: It was replaced throughout the text.*
l.112. Please define aap as “archaeal adhesion pilus” here instead of later. Was the aap pilus added as a separate group only for this reason or based on genomic and structural genomics criteria? There is no evidence that other pili are not retractable.

*A: Aap pili related gene locus shows a very specific gene locus organization. Furthermore, the predicted ATPase structure is substantially different from those of the others, which points to a specific class of surface structures that allow for twitching motility.*

l.124. -127. please write the species names in italics. Figure 1c instead of 1e?

*A:* *This was corrected. For species still classified as Candidatus, only "Ca." is italicized, while the organism name stays non-italic.*

l.137. Please be more specific: what is the name of this ATPase family? Which bacterial secretion systems?

*A: The ATPase family was specified and added.*
l.142. it is not obvious that ATPase tree mirrors the T4F group clustering. The color code in Figure 2a is difficult to see for some groups, notably for Aap, because of overlapping black lines. Also, the archaellum ATPases (orange) appear split in two groups – please comment. It would be helpful to label the branches directly on the tree.

*A: Tree labeling was changed to color-stripes and displayed as a circular rather than unrooted tree, which nicely shows the ATPase classification of the main systems. While some structures appear more closely related, the split in archaellum ATPase clades follows Thermoproteota versus Euryarchaeota lineages with euryarchaeal archaellum ATPases carrying an extended N-terminal region relative to Thermoproteales.*

l.161. This sentence is both incorrect (« ATPase encode for complex N-terminal or C-terminal domains ») and speculative, since the predictions of extra “domains” are of very low pLDDT value and there is no basis to consider them as « complex »- is done!

*A: We agree with the reviewer that the prediction of the extra domains are in low confidence and rephrased the sentence accordingly.*

l.177. why is this an »early specialization » ?

A: We removed the word early as it does not show which system evolved first. Hence, we removed it from the phrasing.

l .178. Figure 2e does not exist.

*A: This should be Figure 2b and we rephrased it accordingly.*

L.188. Examples of arCOG05057 and an S-layer like protein with multiple Ig-like domains are not indicated in Figure 3.

*A: The arCOG05057 was added in Figure 3.*

l.209. name in italics

A: *This was corrected For species still classified as Candidatus, only "Ca." is italicized, while the organism name stays non-italic.*

l.210. does the gene aapX code for the Fe-S cluster protein? It would be helpful if these components were annotated on Figure 4b, like for the archaellum.

*A: We agree with the reviewer and annotated the Figure as for the archaellum. We added further information which one is the platform protein, ATPase. Pilin and accessory components.*
L. 232. What exactly is highlighting diverse surface structures?

*A: We clarified the sentence and rephrased it.*
l.235. ….one transcriptional unit. It remains unclear from this description that the 11-gene cluster does not include the genes encoding the ATPase and the platform protein.

*A: We rephrased the sentence to clarify. The 11-gene cluster contains ATPases and platform proteins, as indicated in the corresponding figure.*

l.237. ….removing minor pilins decreases surface pili (or piliation)

*A: This was removed.*
l.239-240. Please explain « Standalone pilins at a separate locus are organized with the subsystem 1 pilus «

*A: Genes encoding pilins are not found within the main system 1 gene however are likely still part of system 1. These standalone pilins were shown in M. maripaludis to be processed by EppA, and their deletion led to nonpiliated cells (10.1371/journal.pone.0083961). Hence, these pili are part of system 1 despite their separate gene locus.*

l.244. please remove the comma from this sentence.
L. 250. « These observations suggest that multiple TFF of system 2 loci may coexist in a single genome » . Rather : « we show that multiple TFF of system 2 loci coexist in a single genome « .

*A: Both were corrected as suggested.*

L. 257. “ the platform protein is a single gene” – please correct the sentence. Also, please comment on this phenomenon? Is this a result of an initial gene duplication and fusion, or gene fission as proposed by Denise et al. (2019).

*A: We rephrased it accordingly. This is a result of gene fission as already shown in Denise et al., 2019 and we refer to this publication.*

l.262. “Functional specialization of system 2 pili beyond merely forming filaments”. This is a misleading statement. Surely none of the T4F is “merely forming filaments” and each filament has specific surface properties and dynamics which determine their function. Therefore, each one is functionally specialized even in the absence of prominent extra domains/pilins.
Why are pilins or archaellins indicated in figure S2c?

*A: We agree with the reviewer as rephrased it accordingly. In Figure S2C this was a mistake and was changed to pilins.*

279. “The minimal subsystem 4A has two platform proteins ». Please be precise: two genes encoding the platform protein but not two platform proteins. The platform complex has a similar oligomeric architecture whether it is encoded by one or two genes.

*A: We rephrased it accordingly.*

l.290. remove “up and downstream”, as redundant with “flanking”.
l.291. should write: arCOG07037 member near the major pilin gene-
l.292. several Ig-fold domains.

*A: As suggested, these were rephrased.*
L.391. -392. Are the new substrate binding proteins associated only with class 4I systems or are they using other T4F systems for function?

A: other system could be used too though functional association was shown for system 4I in Sulfolobales (doi: 10.1007/s00792-010-0353-0;doi: 10.1111/j.1365-2958.2007.05697.x). It awaits further experimental data to see if other system could serve as a T4F basis too.

Figure 5b. please indicate the prepilin peptidase cleavage site in the weblogo.

*A: This was indicated in Figure 5 as a red box highlighting the cleavage site.*

Other minor comments:
Please be consistent with using capitals in naming T4F structures like Ups, Aap etc.
Figure 1a, separate subsystem from numbers for 4E to 4I .

A: We agree with the reviewer and checke and corrected it in the manuscript.

Reviewer #2: This manuscript describes the results of a large-scale screen for type 4 filament (TFF) gene clusters in Archaeal species. Based on the phylogenetic analyses, the authors group the TFFs into a large number of distinct systems/subsystems, and they then describe details of the similarities and differences among all systems. Overall, the work is well-organized and has a logical flow and, in general, clearly delineates distinct features of each T4SS class. The authors highlight distinct features of the conserved ATPases in each system, speculating that sequence/domain variability has evolved to accommodate other system-specific structural or subunit motifs. The authors also carry out a high-throughput genomics screen for proteins bearing features of TFF pilins, e.g., SPIII cleavage sites and N-terminal alpha-helical TM domains. They report the identification of genes encoding many previously-undescribed pilin-like proteins with new substrate-binding activities in archaeal genomes, speculating that these proteins are incorporated into TFF systems for functional diversification. The strengths of this work are the systematic identification and grouping of TFFs among all the available archaeal genomes in the public repository and the detailed descriptions of the subunit compositions of each system. This feature of the work should serve as a useful resource for scientists studying the structures and broad biological functions of these fascinating machines in bacteria and archaea. However, major weaknesses exist, in particular, speculations that the i) variable domains associated with each of the ATPases confer system-specific functions and ii) newly-identified pilin-like proteins are incorporated into TFFs for novel functionalization. As there is no experimental support for such proposals, the authors need to tone down conclusions from their studies, reserving all conclusions to descriptions of the major conserved and distinctive features of each system. They should make it clear that any structural/sequence variations of core components, while important for classification, may or may not impart the associated TFF with functional diversification. Moreover, they should emphasize the point that, although they have identified many new genes whose products might encode pilin-like subunits or adhesins that incorporate into TFFs, whether they in fact do requires further investigation. Other comments appear below.

*A: We thank the reviewer for the extensive and positive comment!*
Comments:
1. The introduction could be divided into paragraphs, with the last succinctly summarizing the goals/findings of the study – this would strengthen the manuscript and help draw the readership in.

2. It’s difficult to understand the bases for all the distinct subclassifications of the archaeal TFFs other than the two major ones (archaellum, aap-pilus). Can the authors devise a Table summarizing the details of each of the different systems/subsystems, with columns describing the conserved and sequence-specific components, distinctions among the core components of predicted structural or functional importance, the biological activities of each system/subsystem, and references for all papers that have focused on the given systems/subsystems. This unifying table would help the reader tremendously and would be a useful resource for future investigations.

A: *We agree with the reviewer and find it beneficial for the manuscript. This table was added as a supplementary table1 that summarizes the main ArCOGs per system with details on subsystem and key functional importance. Furthermore supplementary table 2 summarizes the mandatory components of a T4F with its corresponding arCOGS and while supplementary table 3 lists arCOGs specific to each subsystem.*

3. L. 92. This isn’t a sentence

4. L. 93. This also isn’t a sentence.

*A: Both were rephrased.*
5. L. 93. I don’t see a supplementary table 1 in the reviewer files.-check or add the excel as supplement.

*A: We added the supplementary now with the revised manuscript.*
6. L. 99. The authors should clarify how this was done more precisely, as apparently the ordering of replicons of the different TFFs forms the basis for differentiating the different subsystems (if this is not the case, the authors need to better describe how they grouped newly identified TFF gene clusters into one of the different systems/subsystems.

*A: This is now explained better in the revised manuscript and described accordingly. Grouping follows Makarova et al., 2016, who defined the systems based on the difference clustering of ATPases, with genes in the neighborhood considered part of the system.*
7. L. 103. Fig. 1C. The sentence isn’t well-structured, and it isn’t clear why Fig. 1C is referenced here, or more broadly why it even exists within Fig. 1.

*A: This sentence was rephrased for clarity.*
8. L. 105. Fig. 1D. In fact, most genomes encode 0 TFFs, and then the second most is 2 TFFs, with 1 TFF being third most abundant. -done

*A: This was rephrased accordingly.*

9. L. 149. Although this analysis identifies phylogenetic variation among the TFF ATPases, it’s a stretch to state that it points to evolutionary diversification driven through incorporation of accessory components. While possible, it is also possible the specific omains or structural motifs play other roles, such as modulating ATPase activity, sensing other cellular signals through interactions with other regulatory proteins to modulate function, stabilize the protein, or they simply were acquired and have no special function.

*A: We agree that stating that the ATPases are a driver of diversification may be too strong a claim. What is clear, however, is that these ATPases are divergent despite conservation and remarkable similarity of the overall machinery. This divergence could indeed arise from the incorporation of additional interaction sites accommodating for novel functions. This could point toward its diversification. As the ATPase is the core component of the T4F and necessary for assembly and function, any structural changes to the ATPase must impact on the overall function and structure of the whole T4F system. As this is highly speculative, we have rephrased the sentence and toned down the final conclusion.*

10. L. 156. Fig. 2. The Fig. shows Sacharalobus solfataricus (not Sulfolobus acidocaldarius as stated in the text) and Haloferzx volcanii.

*A: Is changed.*

11. L. 162. Again, this inference may or may not be true and the statement should be deleted. Same with L. 164 – it’s complete speculation that domains not present in minimal ATPases interfere with or replace a potential rotary mechanism… Delete this statement.

*A: The statements were adapted and toned down as this is hypothetical.*

12. Fig. 2B. The monomer structures – the authors should identify and label conserved structural motifs and the variable ones. Assuming that the latter correspond to the structures colored in blue or grey, it is conceivable that these are just extensions that these proteins have acquired for no functional or structural purpose.

*A: We agree with the reviewer and changed the ATPases labelling to conserved and variable domains.*

13. Fig. 2B. All of these ATPases are predicted to assemble/function as homohexamers, correct? If so, Alphafold-modeled hexameric structures should be very informative, more so than the monomeric structures in generating better predictive power regarding the functional or structural importance of N- or C-terminal extensions.

*A: We added a supplementary figure with the hexameric prediction of the ATPases. These form hexamers, and the variable domains are not a hindrance to that. This points towards its predicted function as ATPases in the T4F systems.*

14. L. 188. Fig. 3 and subsequent figures. It’s often difficult to relate the text to the figure because the figure lacks descriptors used in the text. For example, the text mentions arCOG05057 – this is found nowhere in the figure, even though the figure is cited in this sentence. Also, it’s great that the gene locus and machine figures are color-coded, but interpretation of the figures would be further enhanced by identifying the core genes in the schematics, e.g., ATPase, pilin, platform protein, by name. These names could be appended above the gene schematics as well as next to the components in the machine cartoons. For other figures, genes/proteins specific to the given system should also be labeled if they have a name, e.g., Fe-S cluster domain protein, EpdA, EpdD pilin, adhesin-like domain protein, PilY, etc. Addition of these names would greatly help interpretations of the text and associated figures.

*A: We agree with the reviewer and added additional labels for the core genes.*

15. L. 200 Fig. 4. Why isn’t arCOG03872 also shown in the figure since the text states that

*A:This was added to the figure.*

16. L. 213. That these systems represent simplified pilus systems with these functions: why do these features suggest they are more widespread across Archaea than previously recognized? This observation is followed by unsupported speculation.

A: rephrased

17. L. 229. This is not a sentence, but more importantly, it is not evident why additional pilins with these features suggest another system 1 pilus is likely expressed under certain conditions – another unsupported speculation.

*A: This has been speculated in the paper that identified the additional pilins (doi:* *10.1371/journal.pone.0083961), though experimental proof is lacking. Hence, we rephrased the sentence.*

18. L. 232. Supp. Fig. 1. EpdA should be labeled both in the gene schematic (a) and machine cartoon (b), as should the Epd pilin subunit gene/protein and the adhesin-like domain protein and SPIII protein with a galactose binding domain. Also, the gene schematic (a) shows many more than 11 genes, which ones are the 11 required for pilus production, these should be highlighted in some way.

*A: The figures was changed as suggested.*

19. L. 253. Is there a MinD homolog in the gene schematics shown in (a)? If so, it should be labeled, if not, why is it not there?

*A: It is not shown because, although a MinD homolog was speculated by Makarova et al., no MinD homolog is found within the gene neighborhood. A homolog located elsewhere in the genome might still play a functional role, but it is not directly part of the gene locus for this system.*

20. L. 256. Do the hexameric models of the system 1 vs 2 ATPases better highlight the possible functional/structural importance of the N- and C-terminal extensions of the system 2 ATPases? If so, this should be shown..

*A:We added a supplementary figure with the hexameric predictions. All ATPases form hexamers, though the domains are arranged differently around them. This arrangement could look different in vivo with the additional proteins present, and might form potential interaction sites. The functional importance of these domains is supported by their accessibility for interaction with other T4F system components, though how this occurs needs to be shown experimentally which goes beyond the scope of this manuscript.*

21. L. 268. Please remind the reader what the basis was for assigning gene clusters to system 4 and all of its subsystems – this could simply involve referring the reader to the suggested Table 1 that summarizes the features of all TFF systems/subsystems.

*A: Systems were assigned based on the classification of Makarova et al., 2016, which generated and sorted specific arCOGs into specific subsystems of archaeal T4F. This is summarized in the supplementary table, as suggested.*

22. L. 281. Suppl Fig. 3. Why aren’t arCOG11238 and arCOG06117 presented in the gene schematics and cartoon, given that they are the signature 4A specific pilins? They should be named in these figure panels. Same for the PilY homolog, this should be shown since it is apparently a 4A signature. Also, the sentence beginning on L. 280 is not a sentence, rephrase.

*A: The sentence was rephrased and the figure was changed accordingly.*

23. L. 304. Suppl. Fig. 4. What does the hexameric model show for N- and C-terminal extensions of the ATPase?

*A: A supplementary figure of select ATPases with N- and C-terminal domains was added and shows that these domains do not hamper the formation of hexamers. They are arranged around it and remain accessible as potential interaction sites with other components.*

24. L. 319. Suppl. Fig 5d-f is misnamed – it’s Fig. 4d-f. Also, couldn’t this just be a nonfunctional remnant of another TFF system?

*A: The figures reference was corrected. This could be the case, though the core structural part (the ATPase and platform protein) does look different from that of the other systems. Despite the lack of a pilin-encoding gene, the gene neighborhood seems to be conserved, which points toward a specific subsystem.*

25. L. 324, 326, 335. All these are references to Suppl Fig. 5, not 6.
26. L. 342. This is a reference to Suppl. Fig. 6, not 7.

*A: Both were corrected.*
27. L. 355. Supp. Fig. 7. The UpsX gene/protein should be shown/named as UpsX. Also, in addition to the helicase, the neighboring endonuclease and glycosyltransferase should be shown/named.

*A: Figure labels were added.*
28. L. 363. basEF should be named/shown in the gene schematic.

*A: Figure labels were added.*
29 L. 384. What evidence is there that substrate binding proteins that are not encoded within the TFF gene cluster or that lack SPIII cleavage sites actually associate with TFFs? It would seem this would need to be demonstrated for every substrate binding protein, in particular those with genes clustering together with ABC transporter gene clusters, as these may instead be part of the ABC machinery.

30. L. 395. Given that about a quarter of the identified putative binding proteins are encoded by genes in genomes that lack a TFF locus, the predictive power is quite weak in attempts to ascribe newly-identified putative binding proteins to be novel components of TFFs.

31. L. 408. Although this is an interesting possibility, it remains highly speculative, especially given that these genes are found within ABC transporter clusters and not TFF clusters. Also, there is no information that the proteins identified in Fig. 5d are encoded in genomes that carry TFFs. Overall, the data presented simply do not present a compelling case in support of this proposal.

*A: We agree with the three comments of the reviewer. Association with a T4F locus, if present, needs to be shown experimentally and further validated with functional assays, which is out of the scope of this manuscript. Hence, we have rephrased this part and toned down our claims accordingly.*

32. L. 439. Again, this might be better represented with hexameric models of the ATPases.

*A: This was added now as a supplementary Figure.*

33. L. 443. This isn’t obvious, there are other explanations for the acquisition of the extra sequences/domains.

*A: We agree and rephrased the sentence accordingly.*

34. L. 452. Again, further experiments are needed to support any speculations concerning capacity of TFFs to functionally diversify through incorporation of newly-identified substrate binding proteins.

*A: We agree with the reviewer that further experiments are needed to support these speculations regarding the capacity of T4F to functionally diversify through the incorporation of newly identified substrate binding proteins. Hence, we have rephrased this part and toned down our claims accordingly.*

Reviewer #3: The manuscript “Diversification and functional expansion of archaeal TFF machineries” provides a detailed description of type 4 filament (TFF) systems identified in over 16000 archaeal genomes, including recently discovered archaeal lineages, including major phyla such as Asgards (Promethearchaeati) and DPANN (Nanobdellati). This study represents a major update of a previous analysis of archaeal TFFs published in 2016 (ref. 16). Importantly, the authors built HMM profiles and feature models for MacSyFinder2 software, allowing easy and reliable identification of these systems in newly sequenced archaeal genomes. I have only a few suggestions and comments, which I hope will allow authors to improve the clarity of the text.
1. Please read the entire text carefully again, because some sentences are grammatically correct but unclear. For example: lines 89-95; line 152 (ATP binding motifs, not ATPase binding motifs); avoid using “deep branching euryarchaeota”, be specific; lines 238-239; lines 341 “systems encodes” correct to “systems consist of” or “system loci encode”; avoid using word “prokaryal”; line 380 – “about 7?” how many exactly; “exemplary” in most cases is better be replaced to “a representative” or “selected”, etc.

*A: We thank the reviewer for the comment and suggestions! The revised manuscript was corrected of grammatical errors and typos.*
2. Please provide a better description of the methods:
a. How exactly were HMMs built, what is the source of the alignments?

*A: HMMs were not built by us. Instead, arCOGs corresponding to the systems were pulled from the eggNOG database according to Makarova et al., 2016. MacSyFinder models for each subsystem were then built using these arCOGs, with the ATPase, platform protein, and peptidase set as mandatory components, while the remaining components were set as accessory and required to be within the genetic neighborhood in an ordered replicon.*

b. Cite the most recent arCOG paper (PMID: 30710061)

*A: The recent arCOG paper was added.*
c. How were the feature models built? Give more details, maybe using some examples. If they are described in the supplement, make it clear in both methods and the respective supplementary figure legends

*A: A supplementary table with arcgos were subsystem was added and the model building was described more comprehensively in the methods section.*
d. Explain how potential N-termini containing a Tat secretion sequence or a lipobox were screened. Provide a reference for the software used (if any).

*A: An in-house script was used to look for the Tat motif, containing the RR, and the lipobox motif as published (doi: 10.1038/s41467-025-63625-6). These potential hits, of course, still need to be validated.*
3. Follow current taxonomy terms or make a disclaimer that you are using “old” terminology, and if so, provide correspondence with current terms.

*A: We refer to the current taxonomy and changed it accordingly.*
4. Be consistent with system and subsystem naming – they are described as clades in the supplement. Also, Aap pili were described before as a subsystem 4a, so please mention this.

*A: We have revised the manuscript to ensure consistent naming of systems and subsystems throughout, and clarified that these correspond to the clades described in the supplement. We also now mention that Aap pili were previously described as subsystem 4a.*
5. Check if the arCOG numbers indicated in Figure 3a are specific to the archaellum. I believe only arCOG01817 and arCOG01809 correspond to the ATPase and platform protein, respectively. Double-check other genes and systems too.

*A: It is correct that arCOG01817 and arCOG01809 correspond to the archaellum ATPase and platform protein, respectively. However, to avoid potentially excluding a system, all arCOGs belonging to the ATPase and platform protein families were considered, and the accessory components were then used to define which subsystem each belonged to.*
6. Figure 5d. Consider moving this information to the supplement.

*A: As suggested, this was moved to the supplement.*
7. Consider providing a more detailed description of TFF loci in Asgards and DPANN. Maybe even show these loci in the supplement.

*A: We added a supplementary figure with some exemplary T4F gene loci in DPANN and Asgard archaea. Please note that subsystem classification is difficult, as these genes are not found within a gene locus. System classification and potential function need to be proven experimentally and through structure analysis of the filament architecture.*
