# Supplementary material for: Diversification and functional expansion of archaeal T4F machineries
Source: PLoS Genet. 2026 Oct 14;22(10):e1012139. doi: 10.1371/journal.pgen.1012139 (PMC13705601; doi:10.1371/journal.pgen.1012139)
Supplement: Supplementary file 2 [file pgen.1012139.s018.docx]

REVISION 2

**Reviewer answers for the submitted manuscript “Diversification and expansion of archaeal T4F machineries**
 

**Reviewers' comments:**

Reviewer's Responses to Questions

**Comments to the Authors:
Please note here if the review is uploaded as an attachment.**

Reviewer #1: The authors extensively redacted their manuscript in response to the reviewer’s suggestions.

My comment about Figure 5 and the prepilin peptidase cleavage site remains. The red box highlights the entire conserved region around the cleavage site, which is presumably after residue A/G at position 11. It would be helpful if this site was indicated by an arrow.

*A: We apologize for the oversight and have added a red arrow indicating the cleavage site*.

While I do not have any major objections on the content, the questions of form were not fully addressed, and many details escaped the authors‘ attention. As I believe that PloS Genetics does not engage lectors (copy editors), the remaining minor issues are listed below.

Line 99 remove « for »
Lines 102 and 302-3. please define these non-standard abbreviations when used for the first time.
Lines 104 -105 check for consistent capitals for Ups pilus, Halo, Epd and Aap, Line 319 Ups, and lines 726-728 (figure legend)
Lines 108 and 183. (replace archaeal adhesion pilus by Aap) – since the abbreviation for this pilus has been defined, it should be used thereafter
Line 150-151 this sentence is incomplete
Lines 170-171, 260. please remove « for », encode is sufficient
Line 176 : add « is found » to complete this sentence
Lines 193-194. Here italics are used for candidatus species -please modify to be consistent with the convention as stated in the rebuttal letter.
Line 194. Please use italics for aapX, and for pil1 locus
Line 213. Please use italics for genus and species names Methanobrevibacter and others in lines 266, 286-287, 292, 310, 313-314
Line 217. trancription(al) instead of transcriptomic
Line 229. Replace TFF by T4F
Line 252. remove « are »
Line 262. It is unclear whether there are 2 platform proteins that are flanking the ATPase gene
Line 280. Does this refer to subsystem 4C ? please specify
Line 397.remove “the”
Line 414-415. The second sentence is incomplete please add “they have been”
Line 423-424. This sentence is incomplete and thus unclear
Line 429. ….domain “which” likely arose…
Line 431. …additional domains “which” have evolved…
Figure legends:
Line 708. MacSyFinder
Lines 713 and 714. remove the second T4F
Line 718. Remove “bycolored”
Line 719.correct “regionpart”

The authors should check carefully the final version for typos and misprints (spacing between words is often missing).

*A: We thank the reviewer for highlighting typos and grammtical flaws. We have corrected these and reviewed the manuscript to fix any overlooked errors.*

Reviewer #2: The authors extensively revised their manuscript by carefully addressing my earlier comments and, I believe, the comments of the other reviewers. They toned down some of their conclusions, transitioning from statements of fact to proposals regarding novel features of the different subsystems, and they corrected most of the grammatical flaws. They included additional supplemental tables/files to help the reader understand the distinctions between the different subsystems, and included additional structural predictions, e.g., hexameric forms of the ATPases. Finally, they modified various figures and their corresponding text to better align the two. Overall, the revised manuscript is well-written and provides a comprehensive summary of T4Fs that should prove a useful resource for scientists interested in extracellular surface appendages in archaea as well as bacteria. The authors are encouraged to go through the manuscript once more to identify remaining grammatical flaws (not many) and ensure clear and cohesive sentence/paragraph structure.

*A: We thank Reviewer 2 for the assessment, and we went through the manuscript checking for grammatical errors and sentence structure.*

Reviewer #3: The revised manuscript improved, and the authors made a significant effort to accommodate most of the reviewers’ suggestions, but unfortunately not all.

The first one is taxonomy. There are still “Asgards” (should be Promethearchaeati), DPANN (should be Nanobdellati), Euryarchaea (should be Methanobacteriati), Thaumarchaeota (should be Nitrososphaerota), etc in the text. See my old comments. Make sure that only current terms are used throughout the manuscript and supplement.

*A:We thank the reviewer for the comment. We updated the taxonomy towards the more updated phyla names.*

Second, I asked for a more detailed discussion of T4F machinery in “Asgard” and “DPANN”. Only a few sentences and a reference to Supplementary Figure 10 are included in the discussion in the revised manuscript. But this should be presented in the results section, and more details on what was found there should be reported (which arCOGs were found and with which system they are most compatible).

*A: We agree and have added a new Results subsection ("Scattered T4F-related genes in Prometheoarchaeati and Nanobdellati") that reports this in depth, including supplementary table 4, and 5 listing which arCOGs were found and to which system they are most compatible.*

Also, the discussion of their ATPase positions in the tree should be discussed. Are there any conserved genes that are not recognized by available arCOGs but encoded in those operons? If yes, what are they?

*A: No additional conserved genes were found. The platform protein (ArlJ family), the class III signal peptidase, and the ATPase are the most conserved components across these loci, consistent with Denise et al., 2019 and no further conserved genes outside the existing arCOGs per subsystem were identified. We added this as a sentence in the discussion (l836-839:* *Beyond the core ATPase, platform protein (ArlJ family), and class III signal peptidase, we found no additional genes conserved across T4F loci that are not already described by the existing arCOGs which is consistent with the core set defined by Denise et al. (2019).)*

Third, I am not satisfied with the response to my suggestion about archaellum ATPase and platform protein arCOG numbers. The authors said that to avoid “potentially excluding a system, all arCOGs belonging to the ATPase and platform protein families were considered”. I don’t understand this answer. Do the authors have any data that all these arCOGs are necessary to find all the instances of the archaellum system? Please give examples if this is so. And if this is needed to identify all archaellum systems, all these arCOGs should be indicated for all other T4F systems, because they are homologous, correct? Also, this is not consistent with supplementary table 3 listing archaeallum arCOGs. If needed, consider making your own more specific profile for archaellum core genes.

*A: We thank the reviewer for pushing us to clarify this point, as our previous answer was indeed confusing. To be clear, we are not claiming that all ATPase arCOGs are individually required to find an archaellum locus. Rather, any single member of the ATPase arCOGs is sufficient to retrieve an ATPase related to a T4F, specifically the archaellum locus. This makes arCOG01817 not archaellum-specific but allows to find the most conserved but broadly distributed component of the T4F locus. This means that the mere presence of an ATPase and a platform protein cannot allow to distinguish which T4F system they belong to. It is the gene neighborhood and the accessory components defined per T4F model in the macsyfindermodel that define which subsystem it most likely belongs to. Hence our macsyfindermodels use the arCOGs for platform protein, ATPase and signal peptidase as mandatory components to identify a potential T4F locus. The accessory components per subsystems that are defined for each model based on Makarova et al., 2016 are iteratively searched, and are necessary to assign it to a specific subsystem (see Methods). Our MacSyFinder2 models therefore use the ATPase/platform-protein family (arCOG01817/01818/05609 and the TadC-family platform proteins, respectively) only as broadly defined mandatory components to detect a candidate T4F locus, and rely entirely on the co-localized accessory genes to assign it to a specific subsystem (Methods). This applies identically across all systems, including the archaellum. We agree this distinction was not clear in our original response and have revised the Methods/Results text to state explicitly that the subsystem assignment is neighborhood-based.*
