# Supplementary material for: Diversification and functional expansion of archaeal T4F machineries
Source: PLoS Genet. 2026 Oct 14;22(10):e1012139. doi: 10.1371/journal.pgen.1012139 (PMC13705601; doi:10.1371/journal.pgen.1012139)
Supplement: Peer review history

# Peer review: Decision Letter 0

**DOI:** 10.1371/journal.pgen.1012139.r001

**Authors:** Danielle Garsin, Aretha Fiebig

16 May 2026

PGENETICS-D-26-00397

Diversification and functional expansion of archaeal TFF machineries

PLOS Genetics

Dear Dr. Albers,

Thank you for submitting your manuscript to PLOS Genetics. After careful consideration, we feel that it has merit but does not fully meet PLOS Genetics's publication criteria as it currently stands. Therefore, we invite you to submit a revised version of the manuscript that addresses the points raised during the review process.

Please submit your revised manuscript by Jul 15 2026 11:59PM. If you will need more time than this to complete your revisions, please reply to this message or contact the journal office at plosgenetics@plos.org. When you're ready to submit your revision, log on to https://www.editorialmanager.com/pgenetics/ and select the 'Submissions Needing Revision' folder to locate your manuscript file.

Please include the following items when submitting your revised manuscript:

* A letter that responds to each point raised by the editor and reviewer(s). You should upload this letter as a separate file labeled 'Response to Reviewers'. This file does not need to include responses to any formatting updates and technical items listed in the 'Journal Requirements' section below.

* A marked-up copy of your manuscript that highlights changes made to the original version. You should upload this as a separate file labeled 'Revised Manuscript with Track Changes'.

* An unmarked version of your revised paper without tracked changes. You should upload this as a separate file labeled 'Manuscript'.

If you would like to make changes to your financial disclosure, competing interests statement, or data availability statement, please make these updates within the submission form at the time of resubmission. Guidelines for resubmitting your figure files are available below the reviewer comments at the end of this letter.

As the corresponding author, your ORCID iD is verified in the submission system and will appear in the published article. PLOS supports the use of ORCID, and we encourage all coauthors to register for an ORCID iD and use it as well. Please encourage your coauthors to verify their ORCID iD within the submission system before final acceptance, as unverified ORCID iDs will not appear in the published article. Only the individual author can complete the verification step; PLOS staff cannot verify ORCID iDs on behalf of authors.

We look forward to receiving your revised manuscript.

Kind regards,

Aretha Fiebig, PhD

Academic Editor

PLOS Genetics

Danielle Garsin

Section Editor

PLOS Genetics

Aimée Dudley

Editor-in-Chief

PLOS Genetics

Anne Goriely

Editor-in-Chief

PLOS Genetics

**Additional Editor Comments:**

Your manuscript has been carefully read by three reviewers. The reviewers note that overall the manuscript is well organized and represents a useful update to the classification of archaeal TFF systems. However, they have raised a number of concerns and have offered many suggestions for improvement. In particular, reviewers 1 and 3 raised important concerns about speculative over-interpretation of the data presented. Please carefully consider what conclusions can be drawn from the data and tone down functional speculations that lack supporting experimental evidence.

In addition, all of the reviewers raised concerns about the details of the presentation, both in terms of the writing and the presentation of figures. The reviewers have provided many specific examples in the text that need to be corrected or clarified. Please carefully edit a revised manuscript.

**Journal Requirements:**

1) Please ensure that the CRediT author contributions listed for every co-author are completed accurately and in full.

At this stage, the following Authors/Authors require contributions: Shamphavi Sivabalasarma, and Sonja Albers. Please ensure that the full contributions of each author are acknowledged in the "Add/Edit/Remove Authors" section of our submission form.

The list of CRediT author contributions may be found here: https://journals.plos.org/plosgenetics/s/authorship#loc-author-contributions

2) We have noticed that you have uploaded Supporting Information files, but you have not included a list of legends. Please add a full list of legends for your Supporting Information files after the references list.

3) Please amend your detailed Financial Disclosure statement. This is published with the article. It must therefore be completed in full sentences and contain the exact wording you wish to be published.

State what role the funders took in the study. If the funders had no role in your study, please state: "The funders had no role in study design, data collection and analysis, decision to publish, or preparation of the manuscript.".

If you did not receive any funding for this study, please simply state: u201cThe authors received no specific funding for this work.u201d

**Reviewers' comments:**

Reviewer's Responses to Questions

**Comments to the Authors:**

**Please note here if the review is uploaded as an attachment.**

Reviewer #1: General comments

The article by Sivabalasarma and Albers describes an evolutionary and functional classification of archaeal type IV filament (T4F) nanomachines. This work is an update of an original classification established in 2016 by Makarova, Koonin and Albers (doi: 10.3389/fmicb.2016.00667) which was based on 168 archaeal genome sequences. Based on the sequence analysis of their ATPase motors, the T4F systems were grouped into 4 main clades, with clade 4 branching into 10 different subgroups. In the present manuscript, the authors dispose of a larger sequence database from over 16500 archaeal genomes and use an updated version of the search tool - MacSyFinder2. They added AlphaFold-generated structural predictions on T4F components and construct a structure-based tree of T4F ATPases. The overall system classification remains similar to the previous one with 12 T4F system groups instead of 13 by Makarova et al. They propose that the retractable aap pilus be considered as a separate group, while the previous subgroups 4H and 4J disappear from the classification. The authors demonstrate the diversification of ATPases, with members of some groups acquiring new domains at their extremities. However, structural predictions of these domains are poor and their function remains unknown. The authors also highlight the presence of substrate binding proteins with class III signal peptides, which are widespread presumably beyond the characterised Sulfolobus bindosome systems of group 4I. However, it was not clear whether these binding proteins are connected to different T4F groups and if so which ones.

Overall, despite the availability of a larger database, there are few novel findings and ideas that emerge from this analysis. There is also little discussion on potential evolutionary scenarios. It would have been interesting to discuss the influence of other cellular factors, notably of the cell envelope structure and composition, on the evolution of T4F systems.

Even though the authors used AI tools during writing, the text lacks precision and clarity and is filled with errors. Inconstancies in use of terminology between genes, proteins, COGs are found throughout. A gene does not encode for a protein but either encodes a protein or codes for a protein. Sentences like “the platform protein is a single gene” (l. 258) or “pilins belong to arCOG…” (l.281) are not acceptable for a genetics journal.

Below I provide probably a non-exhaustive list of specific comments:

l.13: Abbreviation TFF is used for type IV filaments. I suggest that T4F would be a little more informative.

l.53. Please provide a reference for this statement about filament subunit gene duplication.

l.56 remove the word “is” at the end of the line.

l.58. a circular statement, to be modified

l.61. as above, change to e.g. “while all machineries share similar core components, specific accessory components are found in each system”

l.64. Makarova et al. describe 13 subsystems, please explain why 11 are mentioned here.

l.67. “….identified several archaeal organisms encoding at least one archaeal TFF system.” Besides having the second “archaeal” word in excess, this sentence is an understatement, as thousands of archaeal genomes have at least one T4F gene cluster.

L. 70. The reference 17 for MacSyFinder should be moved to the end of the previous sentence in line 69.

l.75. Please specify which novel archaeal surface structures were identified here.

l.92. remove “which”.

l.93. the arCOG is an abbreviation for “clusters of orthologous genes”. Please correct.

l. 93-95. please clarify this sentence.

L.103 please remove “in their genomes”.

l. 104. Figure 1c is not appropriately referenced in the text.

l.105. replace “encode for” by “encode” here and throughout.

l.112. Please define aap as “archaeal adhesion pilus” here instead of later. Was the aap pilus added as a separate group only for this reason or based on genomic and structural genomics criteria? There is no evidence that other pili are not retractable.

l.124. -127. please write the species names in italics. Figure 1c instead of 1e?

l.137. Please be more specific: what is the name of this ATPase family? Which bacterial secretion systems?

l.142. it is not obvious that ATPase tree mirrors the T4F group clustering. The color code in Figure 2a is difficult to see for some groups, notably for Aap, because of overlapping black lines. Also, the archaellum ATPases (orange) appear split in two groups – please comment. It would be helpful to label the branches directly on the tree.

l.161. This sentence is both incorrect (« ATPase encode for complex N-terminal or C-terminal domains ») and speculative, since the predictions of extra “domains” are of very low pLDDT value and there is no basis to consider them as « complex ».

l.177. why is this an »early specialization » ?

l .178. Figure 2e does not exist.

L.188. Examples of arCOG05057 and an S-layer like protein with multiple Ig-like domains are not indicated in Figure 3.

l.209. name in italics

l.210. does the gene aapX code for the Fe-S cluster protein? It would be helpful if these components were annotated on Figure 4b, like for the archaellum.

L. 232. What exactly is highlighting diverse surface structures?

l.235. ….one transcriptional unit. It remains unclear from this description that the 11-gene cluster does not include the genes encoding the ATPase and the platform protein.

l.237. ….removing minor pilins decreases surface pili (or piliation)

l.239-240. Please explain « Standalone pilins at a separate locus are organized with the subsystem 1 pilus «

l.244. please remove the comma from this sentence.

L. 250. « These observations suggest that multiple TFF of system 2 loci may coexist in a single genome » . Rather : « we show that multiple TFF of system 2 loci coexist in a single genome « .

L. 257. “ the platform protein is a single gene” – please correct the sentence. Also, please comment on this phenomenon? Is this a result of an initial gene duplication and fusion, or gene fission as proposed by Denise et al. (2019).

l.262. “Functional specialization of system 2 pili beyond merely forming filaments”. This is a misleading statement. Surely none of the T4F is “merely forming filaments” and each filament has specific surface properties and dynamics which determine their function. Therefore, each one is functionally specialized even in the absence of prominent extra domains/pilins.

Why are pilins or archaellins indicated in figure S2c?

279. “The minimal subsystem 4A has two platform proteins ». Please be precise: two genes encoding the platform protein but not two platform proteins. The platform complex has a similar oligomeric architecture whether it is encoded by one or two genes.

l.290. remove “up and downstream”, as redundant with “flanking”.

l.291. should write: arCOG07037 member near the major pilin gene

l.292. several Ig-fold domains.

L.391. -392. Are the new substrate binding proteins associated only with class 4I systems or are they using other T4F systems for function?

Figure 5b. please indicate the prepilin peptidase cleavage site in the weblogo.

Other minor comments:

Please be consistent with using capitals in naming T4F structures like Ups, Aap etc.

Figure 1a, separate subsystem from numbers for 4E to 4I .

Reviewer #2: This manuscript describes the results of a large-scale screen for type 4 filament (TFF) gene clusters in Archaeal species. Based on the phylogenetic analyses, the authors group the TFFs into a large number of distinct systems/subsystems, and they then describe details of the similarities and differences among all systems. Overall, the work is well-organized and has a logical flow and, in general, clearly delineates distinct features of each T4SS class. The authors highlight distinct features of the conserved ATPases in each system, speculating that sequence/domain variability has evolved to accommodate other system-specific structural or subunit motifs. The authors also carry out a high-throughput genomics screen for proteins bearing features of TFF pilins, e.g., SPIII cleavage sites and N-terminal alpha-helical TM domains. They report the identification of genes encoding many previously-undescribed pilin-like proteins with new substrate-binding activities in archaeal genomes, speculating that these proteins are incorporated into TFF systems for functional diversification. The strengths of this work are the systematic identification and grouping of TFFs among all the available archaeal genomes in the public repository and the detailed descriptions of the subunit compositions of each system. This feature of the work should serve as a useful resource for scientists studying the structures and broad biological functions of these fascinating machines in bacteria and archaea. However, major weaknesses exist, in particular, speculations that the i) variable domains associated with each of the ATPases confer system-specific functions and ii) newly-identified pilin-like proteins are incorporated into TFFs for novel functionalization. As there is no experimental support for such proposals, the authors need to tone down conclusions from their studies, reserving all conclusions to descriptions of the major conserved and distinctive features of each system. They should make it clear that any structural/sequence variations of core components, while important for classification, may or may not impart the associated TFF with functional diversification. Moreover, they should emphasize the point that, although they have identified many new genes whose products might encode pilin-like subunits or adhesins that incorporate into TFFs, whether they in fact do requires further investigation. Other comments appear below.

Comments:

1. The introduction could be divided into paragraphs, with the last succinctly summarizing the goals/findings of the study – this would strengthen the manuscript and help draw the readership in.

2. It’s difficult to understand the bases for all the distinct subclassifications of the archaeal TFFs other than the two major ones (archaellum, aap-pilus). Can the authors devise a Table summarizing the details of each of the different systems/subsystems, with columns describing the conserved and sequence-specific components, distinctions among the core components of predicted structural or functional importance, the biological activities of each system/subsystem, and references for all papers that have focused on the given systems/subsystems. This unifying table would help the reader tremendously and would be a useful resource for future investigations.

3. L. 92. This isn’t a sentence.

4. L. 93. This also isn’t a sentence.

5. L. 93. I don’t see a supplementary table 1 in the reviewer files.

6. L. 99. The authors should clarify how this was done more precisely, as apparently the ordering of replicons of the different TFFs forms the basis for differentiating the different subsystems (if this is not the case, the authors need to better describe how they grouped newly identified TFF gene clusters into one of the different systems/subsystems.

7. L. 103. Fig. 1C. The sentence isn’t well-structured, and it isn’t clear why Fig. 1C is referenced here, or more broadly why it even exists within Fig. 1.

8. L. 105. Fig. 1D. In fact, most genomes encode 0 TFFs, and then the second most is 2 TFFs, with 1 TFF being third most abundant.

9. L. 149. Although this analysis identifies phylogenetic variation among the TFF ATPases, it’s a stretch to state that it points to evolutionary diversification driven through incorporation of accessory components. While possible, it is also possible the specific domains or structural motifs play other roles, such as modulating ATPase activity, sensing other cellular signals through interactions with other regulatory proteins to modulate function, stabilize the protein, or they simply were acquired and have no special function.

10. L. 156. Fig. 2. The Fig. shows Sacharalobus solfataricus (not Sulfolobus acidocaldarius as stated in the text) and Haloferzx volcanii.

11. L. 162. Again, this inference may or may not be true and the statement should be deleted. Same with L. 164 – it’s complete speculation that domains not present in minimal ATPases interfere with or replace a potential rotary mechanism… Delete this statement.

12. Fig. 2B. The monomer structures – the authors should identify and label conserved structural motifs and the variable ones. Assuming that the latter correspond to the structures colored in blue or grey, it is conceivable that these are just extensions that these proteins have acquired for no functional or structural purpose.

13. Fig. 2B. All of these ATPases are predicted to assemble/function as homohexamers, correct? If so, Alphafold-modeled hexameric structures should be very informative, more so than the monomeric structures in generating better predictive power regarding the functional or structural importance of N- or C-terminal extensions.

14. L. 188. Fig. 3 and subsequent figures. It’s often difficult to relate the text to the figure because the figure lacks descriptors used in the text. For example, the text mentions arCOG05057 – this is found nowhere in the figure, even though the figure is cited in this sentence. Also, it’s great that the gene locus and machine figures are color-coded, but interpretation of the figures would be further enhanced by identifying the core genes in the schematics, e.g., ATPase, pilin, platform protein, by name. These names could be appended above the gene schematics as well as next to the components in the machine cartoons. For other figures, genes/proteins specific to the given system should also be labeled if they have a name, e.g., Fe-S cluster domain protein, EpdA, EpdD pilin, adhesin-like domain protein, PilY, etc. Addition of these names would greatly help interpretations of the text and associated figures.

15. L. 200 Fig. 4. Why isn’t arCOG03872 also shown in the figure since the text states that these pili invariably contain two pilin homologs.

16. L. 213. That these systems represent simplified pilus systems with these functions: why do these features suggest they are more widespread across Archaea than previously recognized? This observation is followed by unsupported speculation.

17. L. 229. This is not a sentence, but more importantly, it is not evident why additional pilins with these features suggest another system 1 pilus is likely expressed under certain conditions – another unsupported speculation.

18. L. 232. Supp. Fig. 1. EpdA should be labeled both in the gene schematic (a) and machine cartoon (b), as should the Epd pilin subunit gene/protein and the adhesin-like domain protein and SPIII protein with a galactose binding domain. Also, the gene schematic (a) shows many more than 11 genes, which ones are the 11 required for pilus production, these should be highlighted in some way.

19. L. 253. Is there a MinD homolog in the gene schematics shown in (a)? If so, it should be labeled, if not, why is it not there?

20. L. 256. Do the hexameric models of the system 1 vs 2 ATPases better highlight the possible functional/structural importance of the N- and C-terminal extensions of the system 2 ATPases? If so, this should be shown..

21. L. 268. Please remind the reader what the basis was for assigning gene clusters to system 4 and all of its subsystems – this could simply involve referring the reader to the suggested Table 1 that summarizes the features of all TFF systems/subsystems.

22. L. 281. Suppl Fig. 3. Why aren’t arCOG11238 and arCOG06117 presented in the gene schematics and cartoon, given that they are the signature 4A specific pilins? They should be named in these figure panels. Same for the PilY homolog, this should be shown since it is apparently a 4A signature. Also, the sentence beginning on L. 280 is not a sentence, rephrase.

23. L. 304. Suppl. Fig. 4. What does the hexameric model show for N- and C-terminal extensions of the ATPase?

24. L. 319. Suppl. Fig 5d-f is misnamed – it’s Fig. 4d-f. Also, couldn’t this just be a nonfunctional remnant of another TFF system?

25. L. 324, 326, 335. All these are references to Suppl Fig. 5, not 6.

26. L. 342. This is a reference to Suppl. Fig. 6, not 7.

27. L. 355. Supp. Fig. 7. The UpsX gene/protein should be shown/named as UpsX. Also, in addition to the helicase, the neighboring endonuclease and glycosyltransferase should be shown/named.

28. L. 363. basEF should be named/shown in the gene schematic.

29 L. 384. What evidence is there that substrate binding proteins that are not encoded within the TFF gene cluster or that lack SPIII cleavage sites actually associate with TFFs? It would seem this would need to be demonstrated for every substrate binding protein, in particular those with genes clustering together with ABC transporter gene clusters, as these may instead be part of the ABC machinery.

30. L. 395. Given that about a quarter of the identified putative binding proteins are encoded by genes in genomes that lack a TFF locus, the predictive power is quite weak in attempts to ascribe newly-identified putative binding proteins to be novel components of TFFs.

31. L. 408. Although this is an interesting possibility, it remains highly speculative, especially given that these genes are found within ABC transporter clusters and not TFF clusters. Also, there is no information that the proteins identified in Fig. 5d are encoded in genomes that carry TFFs. Overall, the data presented simply do not present a compelling case in support of this proposal.

32. L. 439. Again, this might be better represented with hexameric models of the ATPases.

33. L. 443. This isn’t obvious, there are other explanations for the acquisition of the extra sequences/domains.

34. L. 452. Again, further experiments are needed to support any speculations concerning capacity of TFFs to functionally diversify through incorporation of newly-identified substrate binding proteins.

Reviewer #3: The manuscript “Diversification and functional expansion of archaeal TFF machineries” provides a detailed description of type 4 filament (TFF) systems identified in over 16000 archaeal genomes, including recently discovered archaeal lineages, including major phyla such as Asgards (Promethearchaeati) and DPANN (Nanobdellati). This study represents a major update of a previous analysis of archaeal TFFs published in 2016 (ref. 16). Importantly, the authors built HMM profiles and feature models for MacSyFinder2 software, allowing easy and reliable identification of these systems in newly sequenced archaeal genomes. I have only a few suggestions and comments, which I hope will allow authors to improve the clarity of the text.

1. Please read the entire text carefully again, because some sentences are grammatically correct but unclear. For example: lines 89-95; line 152 (ATP binding motifs, not ATPase binding motifs); avoid using “deep branching euryarchaeota”, be specific; lines 238-239; lines 341 “systems encodes” correct to “systems consist of” or “system loci encode”; avoid using word “prokaryal”; line 380 – “about 7?” how many exactly; “exemplary” in most cases is better be replaced to “a representative” or “selected”, etc.

2. Please provide a better description of the methods:

a. How exactly were HMMs built, what is the source of the alignments?

b. Cite the most recent arCOG paper (PMID: 30710061)

c. How were the feature models built? Give more details, maybe using some examples. If they are described in the supplement, make it clear in both methods and the respective supplementary figure legends

d. Explain how potential N-termini containing a Tat secretion sequence or a lipobox were screened. Provide a reference for the software used (if any).

3. Follow current taxonomy terms or make a disclaimer that you are using “old” terminology, and if so, provide correspondence with current terms.

4. Be consistent with system and subsystem naming – they are described as clades in the supplement. Also, Aap pili were described before as a subsystem 4a, so please mention this.

5. Check if the arCOG numbers indicated in Figure 3a are specific to the archaellum. I believe only arCOG01817 and arCOG01809 correspond to the ATPase and platform protein, respectively. Double-check other genes and systems too.

6. Figure 5d. Consider moving this information to the supplement.

7. Consider providing a more detailed description of TFF loci in Asgards and DPANN. Maybe even show these loci in the supplement.

**********

**Have all data underlying the figures and results presented in the manuscript been provided?**

Large-scale datasets should be made available via a public repository as described in the *PLOS Genetics*  data availability policy, and numerical data that underlies graphs or summary statistics should be provided in spreadsheet form as supporting information.

Reviewer #1: **No:**  Links to TFF sequence data and structure-based ATPase tree data

Reviewer #2: Yes

Reviewer #3: Yes

**********

PLOS authors have the option to publish the peer review history of their article (what does this mean?). If published, this will include your full peer review and any attached files.

If you choose “no”, your identity will remain anonymous but your review may still be made public.

**Do you want your identity to be public for this peer review?**  For information about this choice, including consent withdrawal, please see our Privacy Policy.

Reviewer #1: No

Reviewer #2: No

Reviewer #3: **Yes:**  Dr. Kira Makarova

[NOTE: If reviewer comments were submitted as an attachment file, they will be attached to this email and accessible via the submission site. Please log into your account, locate the manuscript record, and check for the action link "View Attachments". If this link does not appear, there are no attachment files.]

**Figure resubmission:**

**Reproducibility:**

To enhance the reproducibility of your results, we recommend that authors of applicable studies deposit laboratory protocols in protocols.io, where a protocol can be assigned its own identifier (DOI) such that it can be cited independently in the future. Additionally, PLOS ONE offers an option to publish peer-reviewed clinical study protocols. Read more information on sharing protocols at https://plos.org/protocols?utm_medium=editorial-email&utm_source=authorletters&utm_campaign=protocols

# Author comment: Author response to Decision Letter 1

**DOI:** 10.1371/journal.pgen.1012139.r002

19 Jul 2026

Attachment Submitted filename: Revision_reviewercommentwithanswers.docx

# Peer review: Decision Letter 1

**DOI:** 10.1371/journal.pgen.1012139.r003

**Authors:** Danielle Garsin, Aretha Fiebig

6 Aug 2026

PGENETICS-D-26-00397R1

Diversification and functional expansion of archaeal T4F machineries

PLOS Genetics

Dear Dr. Albers,

Thank you for submitting your manuscript to PLOS Genetics. After careful consideration, we feel that it has merit but does not fully meet PLOS Genetics's publication criteria as it currently stands. Therefore, we invite you to submit a revised version of the manuscript that addresses the points raised during the review process.

Please submit your revised manuscript within by Sep 05 2026 11:59PM. If you will need more time than this to complete your revisions, please reply to this message or contact the journal office at plosgenetics@plos.org. When you're ready to submit your revision, log on to https://www.editorialmanager.com/pgenetics/ and select the 'Submissions Needing Revision' folder to locate your manuscript file.

Please include the following items when submitting your revised manuscript:

* A letter that responds to each point raised by the editor and reviewer(s). You should upload this letter as a separate file labeled 'Response to Reviewers'. This file does not need to include responses to formatting updates and technical items listed in the 'Journal Requirements' section below.

* A marked-up copy of your manuscript that highlights changes made to the original version. You should upload this as a separate file labeled 'Revised Manuscript with Track Changes'.

* An unmarked version of your revised paper without tracked changes. You should upload this as a separate file labeled 'Manuscript'.

If you would like to make changes to your financial disclosure, competing interests statement, or data availability statement, please make these updates within the submission form at the time of resubmission. Guidelines for resubmitting your figure files are available below the reviewer comments at the end of this letter.

As the corresponding author, your ORCID iD is verified in the submission system and will appear in the published article. PLOS supports the use of ORCID, and we encourage all coauthors to register for an ORCID iD and use it as well. Please encourage your coauthors to verify their ORCID iD within the submission system before final acceptance, as unverified ORCID iDs will not appear in the published article. Only the individual author can complete the verification step; PLOS staff cannot verify ORCID iDs on behalf of authors.

We look forward to receiving your revised manuscript.

Kind regards,

Aretha Fiebig, PhD

Academic Editor

PLOS Genetics

Danielle Garsin

Section Editor

PLOS Genetics

Aimée Dudley

Editor-in-Chief

PLOS Genetics

Anne Goriely

Editor-in-Chief

PLOS Genetics

**Additional Editor Comments (if provided):**

Thank you for your revised manuscript expanding and updating the classification of archaeal T4F. The reviews are largely satisfied with your careful responses, although they note a few additional minor editorial changes. Reviewer 1 expressed a few, unresolved concerns. Please include the most current taxonomic names (even parenthetically adjacent to the first use of the more commonly known names). Are there additional comments you could add to the results about T4F in “Asgard” and “DPANN”? This work promises to be a valuable resource for the community. I look forward to a final version.

**Journal Requirements:**

If the reviewer comments include a recommendation to cite specific previously published works, please review and evaluate these publications to determine whether they are relevant and should be cited. There is no requirement to cite these works unless the editor has indicated otherwise.

1) Please amend your detailed Financial Disclosure statement. This is published with the article. It must therefore be completed in full sentences and contain the exact wording you wish to be published.

i) State what role the funders took in the study. If the funders had no role in your study, please state: "The funders had no role in study design, data collection and analysis, decision to publish, or preparation of the manuscript."

ii) If any authors received a salary from any of your funders, please state which authors and which funders..

If you did not receive any funding for this study, please simply state: u201cThe authors received no specific funding for this work.u201d

**Reviewers' comments:**

Reviewer's Responses to Questions

**Comments to the Authors:**

**Please note here if the review is uploaded as an attachment.**

Reviewer #1: The authors extensively redacted their manuscript in response to the reviewer’s suggestions.

My comment about Figure 5 and the prepilin peptidase cleavage site remains. The red box highlights the entire conserved region around the cleavage site, which is presumably after residue A/G at position 11. It would be helpful if this site was indicated by an arrow.

While I do not have any major objections on the content, the questions of form were not fully addressed, and many details escaped the authors‘ attention. As I believe that PloS Genetics does not engage lectors (copy editors), the remaining minor issues are listed below.

Line 99 remove « for »

Lines 102 and 302-3. please define these non-standard abbreviations when used for the first time.

Lines 104 -105 check for consistent capitals for Ups pilus, Halo, Epd and Aap, Line 319 Ups, and lines 726-728 (figure legend)

Lines 108 and 183. (replace archaeal adhesion pilus by Aap) – since the abbreviation for this pilus has been defined, it should be used thereafter

Line 150-151 this sentence is incomplete

Lines 170-171, 260. please remove « for », encode is sufficient

Line 176 : add « is found » to complete this sentence

Lines 193-194. Here italics are used for candidatus species -please modify to be consistent with the convention as stated in the rebuttal letter.

Line 194. Please use italics for aapX, and for pil1 locus (Line 226)

Line 213. Please use italics for genus and species names Methanobrevibacter and others in lines 266, 286-287, 292, 310, 313-314

Line 217. trancription(al) instead of transcriptomic

Line 229. Replace TFF by T4F

Line 252. remove « are »

Line 262. It is unclear whether there are 2 platform proteins that are flanking the ATPase gene

Line 280. Does this refer to subsystem 4C ? please specify

Line 397.remove “the”

Line 414-415. The second sentence is incomplete please add “they have been”

Line 423-424. This sentence is incomplete and thus unclear

Line 429. ….domain “which” likely arose…

Line 431. …additional domains “which” have evolved…

Figure legends:

Line 708. MacSyFinder

Lines 713 and 714. remove the second T4F

Line 718. Remove “bycolored”

Line 719.correct “regionpart”

The authors should check carefully the final version for typos and misprints (spacing between words is often missing).

Reviewer #2: The authors extensively revised their manuscript by carefully addressing my earlier comments and, I believe, the comments of the other reviewers. They toned down some of their conclusions, transitioning from statements of fact to proposals regarding novel features of the different subsystems, and they corrected most of the grammatical flaws. They included additional supplemental tables/files to help the reader understand the distinctions between the different subsystems, and included additional structural predictions, e.g., hexameric forms of the ATPases. Finally, they modified various figures and their corresponding text to better align the two. Overall, the revised manuscript is well-written and provides a comprehensive summary of T4Fs that should prove a useful resource for scientists interested in extracellular surface appendages in archaea as well as bacteria. The authors are encouraged to go through the manuscript once more to identify remaining grammatical flaws (not many) and ensure clear and cohesive sentence/paragraph structure.

Reviewer #3: The revised manuscript improved, and the authors made a significant effort to accommodate most of the reviewers’ suggestions, but unfortunately not all.

The first one is taxonomy. There are still “Asgards” (should be Promethearchaeati), DPANN (should be Nanobdellati), Euryarchaea (should be Methanobacteriati), Thaumarchaeota (should be Nitrososphaerota), etc in the text. See my old comments. Make sure that only current terms are used throughout the manuscript and supplement.

Second, I asked for a more detailed discussion of T4F machinery in “Asgard” and “DPANN”. Only a few sentences and a reference to Supplementary Figure 10 are included in the discussion in the revised manuscript. But this should be presented in the results section, and more details on what was found there should be reported (which arCOGs were found and with which system they are most compatible). Also, the discussion of their ATPase positions in the tree should be discussed. Are there any conserved genes that are not recognized by available arCOGs but encoded in those operons? If yes, what are they?

Third, I am not satisfied with the response to my suggestion about archaellum ATPase and platform protein arCOG numbers. The authors said that to avoid “potentially excluding a system, all arCOGs belonging to the ATPase and platform protein families were considered”. I don’t understand this answer. Do the authors have any data that all these arCOGs are necessary to find all the instances of the archaellum system? Please give examples if this is so. And if this is needed to identify all archaellum systems, all these arCOGs should be indicated for all other T4F systems, because they are homologous, correct? Also, this is not consistent with supplementary table 3 listing archaeallum arCOGs. If needed, consider making your own more specific profile for archaellum core genes.

**********

**Have all data underlying the figures and results presented in the manuscript been provided?**

Large-scale datasets should be made available via a public repository as described in the *PLOS Genetics*  data availability policy, and numerical data that underlies graphs or summary statistics should be provided in spreadsheet form as supporting information.

Reviewer #1: Yes

Reviewer #2: Yes

Reviewer #3: Yes

**********

PLOS authors have the option to publish the peer review history of their article (what does this mean?). If published, this will include your full peer review and any attached files.

If you choose “no”, your identity will remain anonymous but your review may still be made public.

**Do you want your identity to be public for this peer review?**  For information about this choice, including consent withdrawal, please see our Privacy Policy.

Reviewer #1: No

Reviewer #2: No

Reviewer #3: No

[NOTE: If reviewer comments were submitted as an attachment file, they will be attached to this email and accessible via the submission site. Please log into your account, locate the manuscript record, and check for the action link "View Attachments". If this link does not appear, there are no attachment files.]

**Figure resubmission:**

**Reproducibility:**

To enhance the reproducibility of your results, we recommend that authors deposit laboratory protocols in protocols.io, where a protocol can be assigned its own identifier (DOI) such that it can be cited independently in the future. Additionally, PLOS ONE offers an option to publish peer-reviewed clinical study protocols. Read more information on sharing protocols at https://plos.org/protocols?utm_medium=editorial-email&utm_source=authorletters&utm_campaign=protocols

# Author comment: Author response to Decision Letter 2

**DOI:** 10.1371/journal.pgen.1012139.r004

23 Sep 2026

Attachment Submitted filename: revision_2reviewercomment.docx

# Editor report: Decision Letter 2

**DOI:** 10.1371/journal.pgen.1012139.r005

**Authors:** Danielle Garsin, Aretha Fiebig

30 Sep 2026

Dear Dr Albers,

We are pleased to inform you that your manuscript entitled "Diversification and functional expansion of archaeal T4F machineries" has been editorially accepted for publication in PLOS Genetics. Congratulations!

Before your submission can be formally accepted and sent to production you will need to complete our formatting changes, which you will receive in a follow up email. Please be aware that it may take several days for you to receive this email; during this time no action is required by you. Please note: the accept date on your published article will reflect the date of this provisional acceptance, but your manuscript will not be scheduled for publication until the required changes have been made.

Once your paper is formally accepted, an uncorrected proof of your manuscript will be published online ahead of the final version, unless you’ve already opted out via the online submission form. If, for any reason, you do not want an earlier version of your manuscript published online or are unsure if you have already indicated as such, please let the journal staff know immediately at plosgenetics@plos.org.

In the meantime, please log into Editorial Manager at https://www.editorialmanager.com/pgenetics/, click the "Update My Information" link at the top of the page, and update your user information to ensure an efficient production and billing process. Note that PLOS requires an ORCID iD for all corresponding authors. Therefore, please ensure that you have an ORCID iD and that it is validated in Editorial Manager. To do this, go to ‘Update my Information’ (in the upper left-hand corner of the main menu), and click on the Fetch/Validate link next to the ORCID field.  This will take you to the ORCID site and allow you to create a new iD or authenticate a pre-existing iD in Editorial Manager.

If you have a press-related query, or would like to know about making your underlying data available (as you will be aware, this is required for publication), please see the end of this email. If your institution or institutions have a press office, please notify them about your upcoming article at this point, to enable them to help maximise its impact. Inform journal staff as soon as possible if you are preparing a press release for your article and need a publication date.

Thank you again for supporting open-access publishing; we are looking forward to publishing your work in PLOS Genetics!

Yours sincerely,

Aretha Fiebig, PhD

Academic Editor

PLOS Genetics

Danielle Garsin

Section Editor

PLOS Genetics

Aimée Dudley

Editor-in-Chief

PLOS Genetics

Anne Goriely

Editor-in-Chief

PLOS Genetics

www.plosgenetics.org

BlueSky: @plos.bsky.social

----------------------------------------------------

Comments from the reviewers (if applicable):

Thank you for your attention to the final reviewer feedback, and for your submission of this comprehensive analysis of archaeal type 4 filament machinery to PLOS Genetics.

----------------------------------------------------

**Data Deposition**

If you have submitted a Research Article or Front Matter that has associated data that are not suitable for deposition in a subject-specific public repository (such as GenBank or ArrayExpress), one way to make that data available is to deposit it in the Dryad Digital Repository. As you may recall, we ask all authors to agree to make data available; this is one way to achieve that. A full list of recommended repositories can be found on our website.

The following link will take you to the Dryad record for your article, so you won't have to re‐enter its bibliographic information, and can upload your files directly:

http://datadryad.org/submit?journalID=pgenetics&manu=PGENETICS-D-26-00397R2

More information about depositing data in Dryad is available at http://www.datadryad.org/depositing. If you experience any difficulties in submitting your data, please contact help@datadryad.org for support.

Additionally, please be aware that our data availability policy requires that all numerical data underlying display items are included with the submission, and you will need to provide this before we can formally accept your manuscript, if not already present.

----------------------------------------------------

**Press Queries**

If you or your institution will be preparing press materials for this manuscript, or if you need to know your paper's publication date for media purposes, please inform the journal staff as soon as possible so that your submission can be scheduled accordingly. Your manuscript will remain under a strict press embargo until the publication date and time. This means an early version of your manuscript will not be published ahead of your final version. PLOS Genetics may also choose to issue a press release for your article. If there's anything the journal should know or you'd like more information, please get in touch via plosgenetics@plos.org.

# Editor report: Acceptance letter

**DOI:** 10.1371/journal.pgen.1012139.r006

**Authors:** Danielle Garsin, Aretha Fiebig

PGENETICS-D-26-00397R2

Diversification and functional expansion of archaeal T4F machineries

Dear Dr Albers,

We are pleased to inform you that your manuscript entitled "Diversification and functional expansion of archaeal T4F machineries" has been formally accepted for publication in PLOS Genetics! Your manuscript is now with our production department and you will be notified of the publication date in due course.

The corresponding author will soon be receiving a typeset proof for review, to ensure errors have not been introduced during production. Please review the PDF proof of your manuscript carefully, as this is the last chance to correct any errors. Please note that major changes, or those which affect the scientific understanding of the work, will likely cause delays to the publication date of your manuscript.

Soon after your final files are uploaded, unless you have opted out or your manuscript is a front-matter piece, the early version of your manuscript will be published online. The date of the early version will be your article's publication date. The final article will be published to the same URL, and all versions of the paper will be accessible to readers.

For Research Articles, you will receive an invoice from PLOS for your publication fee after your manuscript has reached the completed accept phase. If you receive an email requesting payment before acceptance or for any other service, this may be a phishing scheme. Learn how to identify phishing emails and protect your accounts at https://explore.plos.org/phishing.

Thank you again for supporting PLOS Genetics and open-access publishing. We are looking forward to publishing your work!

With kind regards,

Sharmila Kamatchi

PLOS Genetics

On behalf of:

The PLOS Genetics Team

Carlyle House, Carlyle Road, Cambridge CB4 3DN | United Kingdom

plosgenetics@plos.org | +44 (0) 1223-442823

plosgenetics.org | Twitter: @PLOSGenetics
